# Enhancer RNA promotes resistance to radiotherapy in bone-metastatic prostate cancer by m^6^A modification

**DOI:** 10.7150/thno.78687

**Published:** 2023-01-01

**Authors:** Yu Zhao, Simeng Wen, Hang Li, Chun-Wu Pan, Yulei Wei, Ting Huang, Zhaochen Li, Yinhui Yang, Saijun Fan, Yingyi Zhang

**Affiliations:** 1 Tianjin Key Laboratory of Radiation Medicine and Molecular Nuclear Medicine, Institute of Radiation Medicine, Chinese Academy of Medical Sciences and Peking Union Medical College, Tianjin, 300192, China; 2 Department of Urology, The Second Hospital of Tianjin Medical University, Tianjin Medical University, Tianjin, 300211, China; 3 Department of Urology, Renji Hospital, School of Medicine, Shanghai Jiao Tong University, Shanghai, 200127, China; 4 Department of Gynecology and Obstetrics, Tianjin First Central Hospital, Tianjin, 300192, China; 5 Department of Urology, Shanghai Changhai Hospital, Naval Medical University, Shanghai, 200433, China; 6 Department of Biochemistry and Molecular Biology, Mayo Clinic, Rochester, MN 55905, USA

**Keywords:** Bone metastatic prostate cancer, m^6^A, Enhancer RNA, m^6^Am, Radiotherapy.

## Abstract

**Rationale:** Prostate cancer metastasizes to the bone with the highest frequency and exhibits high resistance to ^177^Lu-prostate-specific membrane antigen (PSMA) radioligand therapy. Little is known about bone metastatic prostate cancer (mPCa) resistance to radiation.

**Methods:** We filtered the metastatic eRNA using RNA-seq, MeRIP-seq, RT-qPCR and bioinformation. Western blot, RT-qPCR, CLIP, co-IP and RNA pull-down assays were used for RNA/protein interaction, RNA or protein expression examination. MTS assay was used to determine cell viability in vitro, xenograft assay was used to examine the tumor growth in mice.

**Results:** In this study, we screened and identified bone-specific N6 adenosine methylation (m^6^A) on enhancer RNA (eRNA) that played a post-transcriptional functional role in bone mPCa and was correlated with radiotherapy (RT) resistance. Further data demonstrated that RNA-binding protein KHSRP recognized both m^6^A at eRNA and m^6^Am at 5'-UTR of mRNA to block RNA degradation from exoribonuclease XRN2. Depletion of the *MLXIPe*/KHSRP/*PSMD9* regulatory complex inhibited tumor growth and RT sensitization of bone mPCa xenograft *in vitro* and *in vivo*.

**Conclusions:** Our findings indicate that a bone-specific m^6^A-modified eRNA plays a vital role in regulating mPCa progression and RT resistance and might be a novel specific predictor for cancer RT.

## Introduction

Prostate cancer (PCa) is the most common malignancy in males worldwide and is the second-most common cause of cancer death 1, 2. Metastasis is the leading cause of death in prostate cancer, with about 80% of metastasizing to bones, such as the hip, spine, and pelvis. Only 3% of patients with bone metastases survive for five years 1, 3-6. Androgen deprivation therapy is the primary treatment for advanced prostate cancer; however castration-resistant prostate cancer (CRPC), especially metastatic prostate cancer (mPCa), remains a challenge for treatment 7. Radiotherapy (RT) is another critical component of cancer management in over 50% of cancer patients 8, 9. Even though RT shows benefits for high-risk localized advanced PCa, distant mPCa are RT resistant 7, 10, 11. Recent clinical trials showed that androgen deprivation therapy in combination with radiation therapy for bone mPCa did not significantly differ in overall survival compared to androgen deprivation therapy alone 12. The underlying molecular mechanism for RT resistance of bone mPCa cells remains unclear.

Among over 160 types of cellular RNA modifications, N^6^-adenosine methylation (m^6^A) is the most prevalent chemical modification of mRNAs in eukaryotes 13-15. In eukaryotic cells, m^6^A is catalyzed by m^6^A methyltransferases METTL3 and METTL14 and demethylated by FTO and (ALKBH5) 16-18. FTO was identified as the first RNA demethylase that catalyzes the reversal of m^6^A and 2-*O*-dimethyladenosine (m^6^Am) methylation on mRNAs 19. The functional effects of mRNA m^6^A on cellular processes include RNA nuclear transport, secondary structure, splicing, stability and translation efficiency 20-29. However, there is limited information on the m^6^A function in enhancer RNAs (eRNAs).

Tissue-specific or disease-specific gene expression is established primarily by the transcription complexes at enhancers 30, which are critical genomic elements that regulate transcription and are distant from gene promoters 31-33. eRNAs are involved in the activation of gene expression and transcriptional regulation and act as regulators of adjacent elements and complexes in cis and trans modes 34-36. Furthermore, eRNAs interact with cohesion complexes to facilitate promoter and enhancer looping 37. Independent of their function in looping, eRNAs promote RNA polymerase-II (Pol-II) phosphorylation and elongation by activating the p-TEBb complex and removing the NELF complex 38, 39. A recent study reported that eRNA facilitated transcriptional condensate formation through its m^6^A in Erα-related cells 40. Due to the relevance of enhancers in cancer, immune diseases, and neurodegeneration, significant challenges need to be addressed in understanding the mechanisms of enhancers and designing new strategies for enhancer/eRNA targeting and novel therapeutic modalities in cancer medicine. However, the role and modification of eRNAs in cancer are not fully understood.

In this study, we identified a novel m^6^A function of eRNA that was involved in RT resistance in bone mPCa and demonstrated that eRNA m^6^A was associated with poor prognosis of patients with RT. Our data also showed that KHSRP recognized and interacted with m^6^A at* MLXIPe* eRNA and m^6^Am at 5'-UTR of mRNA to block RNA degradation by XRN2. Analysis of the regulatory complex involving eRNA/KHSRP/mRNA might provide new insights into the potential mechanism of RT resistance of bone metastatic prostate cancer. Thus, *MLXIPe* might be a potential predictive marker and therapeutic target for bone-mPCa.

## Materials and Methods

### Plasmids and antibodies

*MLXIPe* eRNA and *KHSRP* were generated by cloning the corresponding cDNAs into pcDNA3.1(+) vector with flag or myc tags designed in the primers. cDNA fragments were amplified by Phusion polymerase (NEB, USA) using Phusion High-Fidelity PCR Master Mix. The insert and deletion mutants were constructed using QuikChange II Site Directed-Mutagenesis Kit (Agilent, USA). Antibodies are shown in Table S4.

### Clinical samples, cell lines, and patient-derived xenograft (PDX)

PCa primary tumors, bone metastases, and adjacent non-tumor tissues were obtained from patients at the Tianjin Second Hospital of Tianjin Medical University, Tianjin, between Jan 1, 2000 and Sep 1, 2020 in accordance with Tianjin Medical University Institutional Review Board approved protocols. Patient specimens were manually dissected using a sterile scalpel blade into 4 mm^3^ pieces and stored in a transport medium at 4 °C until xenograft preparation. Operative tumor tissue was mechanically disaggregated, triturated through a 1 mL syringe, mixed 1:1 with Growth Factor Reduced Matrigel (BD Bioscience, CA, USA) and 200 µL injected into the flank of Athymic Nude mice. Mice were monitored for tumor growth for at least nine months. At the time of surgery, host mice were supplemented with a subcutaneous 5 mm silastic testosterone implant to increase circulating testosterone levels 41. Viable xenografts were propagated by direct serial transplantation for at least three generations before being considered an established xenograft line. Tumor tissue was cryopreserved at initial and subsequent early passages. Short-term explant cultures were obtained from a primary patient-derived bone metastatic prostate panel. PDXs were propagated in 6-8 weeks-old male NOD-SCID mice, and AI xenografts were propagated in SCID mice. Mice were housed in the Tianjin Medical University pathogen-free rodent facility. All procedures were approved by the Tianjin Medical University Institutional Animal Care and Use Committee 42. LNCaP, C4-2B, and PC3 cells were purchased from American Type Culture Collection (ATCC) (Manassas, VA, USA). The C4-2 cell line was obtained from UroCorpoation. LNCaP, C4-2, C4-2B, and PDX-related primary cells were cultured in RPMI 1640 medium supplemented with 10% fetal bovine serum (FBS) or charcoal-stripped, androgen-depleted FBS (Invitrogen) and 100 µg/ml penicillin-streptomycin-glutamine (Invitrogen) at 37ºC with 5% CO_2_. PC3 cells were cultured in a K-12 medium supplemented with 10% FBS or charcoal-stripped FBS. Cell authentication was performed using STR profiling, as previously described 43, 44.

### Positron emission tomography (PET) image acquisition, reconstruction, and analysis

The median injected activity of the ^68^Ga-PSMA-11 (Glu-NH-CO-NH-Lys-(Ahx)-[^68^Ga]) ligand was 198 MBq (5.3 mCi) (range, 107.3-233.1 MBq [2.8-6.4 mCi]), and the median tracer uptake period was 1 h (range, 46-110 min). The images were acquired using a PET/CT scanner (Infinia Hawkeye4; GE) 45. The PET image contained a whole-body scan (pelvis to vertex, 4 min/bed position depending on the patient weight), a dedicated pelvic scan after voiding (same acquisition time/bed position time as used for the whole body), and a dedicated scan of the lower extremities (pelvis to toes, 1 min/bed position for a total of 10 min). The PET images were reconstructed using random-event, attenuation, dead time, and scatter corrections. PET images were reconstructed with an iterative algorithm (ordered-subset expectation maximization) in an axial 168 × 168 matrix on the Biograph 64 TruePoint (2-dimensional, 2 iterations, 8 subsets) and in a 200 × 200 matrix on the Biograph mCT (3-dimensional, 2 iterations, 24 subsets, gaussian filter 5.0). The focal uptake of ^68^Ga-PSMA-11 above the background level and not related to the physiologic uptake or known pitfalls was considered the PSMA-positive signal 45, 46.

### Mouse xenograft generation and tumor growth measurement

For tumor growth under ionizing radiation (IR), 6-week- old NSG male mice were injected with 5 × 10^6^ cancer cells infected with lentivirus or shRNAs and/or expression vectors in 100 μl PBS with 100 μl of Matrigel matrix (BD Bioscience) in one side of flanks. After injection of tumor cells into mice, right flank tumors were radiated by 10 Gy X-ray beam for 14 days and monitored until they reached maximum tumor volumes of 1,000 mm^3^. Subsequently, tumor growth was measured with a caliper every 7 days.

### Biotin-labeled RNA pull-down and Western blot analysis

RNAs were biotin-labeled during transcription using Biotin RNA Labeling Mix (Roche) and T7 polymerase (New England Biolabs). Various deletion mutations within this region were generated by mutagenesis using a KOD-Plus-Mutagenesis Kit (TOYOBO). P-90 primary cells cultured in androgen-depleted medium were lysed in modified binding buffer (150mM NaCl, 50mM Tris-HCl pH7.5, 0.1% SDS 1% Nonidet P-40 (NP-40), and 1% protease inhibitor cocktail). Cell lysates were incubated with biotin-labeled RNAs and streptavidin beads at 4ºC for 10 h. The beads were washed in the wash buffer (150 mM NaCl; 50 mM Tris; pH 7.5; 0.05% NP-40; 1mM MgCl2) at 4ºC six times. The samples were subjected to Western blot analyses as described previously 39. Briefly, protein samples were denatured and subjected to SDS-polyacrylamide gel electrophoresis (SDS/PAGE) and transferred to nitrocellulose membranes (Bio-Rad). The membranes were immunoblotted with specific primary antibodies and horseradish peroxidase-conjugated secondary antibodies and visualized by SuperSignal West Pico Stable Peroxide Solution (Thermo Scientific). The antibodies are shown in Table S4. The original Western blotting data are shown in Figure S7.

### Clustered regularly interspaced short palindromic repeats (CRISPR)-Cas9 system

The CRISPR-Cas9-generated assay was completed using the CRISPR-Cas9 tool kit (Santa Cruz). gRNAs were cloned in lentivirusV2 plasmid under the U6 promoter as previously described 42. The gRNAs are shown in Table S4.

### Cross-linking immunoprecipitation (CLIP)

Primary cells (6 x 10^6^) were treated with 100 µM 4-thiouridine (4SU) for 8 h, washed with cold PBS, and irradiated once with 150 mJ/cm^2^ at 365 nm using a Stratalinker. Cells were lysed in the lysis buffer (100 mM NaCl, 50 mM Tris-HCl pH 7.5, 1% NP-40, 0.1% SDS, 0.5% sodium deoxycholate, protease inhibitor cocktail and RNase inhibitors) with protease inhibitors (1 mL) and transferred to 1.5 mL microtubes. The lysate was partially digested by 2.5 U/µL RNaseT1/A for 15 min at 22^o^C for iCLIP. RNA was immunoprecipitated with Flag, KHSRP, or XRN2 antibodies and protein A/G beads for 10 h at 4^o^C, washed 6 times, RNA was phosphorylated by T4 PNK and ligated between 5' and 3' ends by RNA T4 ligase. SDS-PAGE loading buffer was added, incubated at 65^o^C for 10 min, and the mixture was separated on the SDS-PAGE gel. The RNA-protein complexes were then transferred to a nitrocellulose membrane using a wet transfer apparatus (30V for 1 h). The membrane with the target protein was cut up, and the targeted membrane piece was incubated with proteinase K to de-crosslink, following which RNA was reverse transcribed into cDNA and subjected to real-time qPCR analysis (Yeasen) 42.

### γ-H2AX staining

γ-H2AX staining in PDX primary cells was performed, as described previously 47. Primary cells infected with empty vector (EV) or plasmids expressing *MLXIPe* were treated with 4Gy IR. At each time point after IR, the cells were harvested for immunofluorescence with antibodies for γ-H2AX. Data were calculated from six biological replicates.

### Analysis of publicly available datasets

RNA-seq were deposited in the GEO. The GEO numbers are SRR21733498, SRR21733499, SRR21733500, SRR21733501, SRR21733502, and SRR21733503. m^6^A or m^6^Am related sequences (GSE63753, GSE92375) 48, 49 were obtained from our laboratory and publicly available RNA expression datasets in prostate cancer (GSE55032, GSE137209) 39, 50 using Gene Expression Omnibus (GEO).

### Statistical analysis

Statistical analysis was performed by GraphPad Prism7 and R scripts. Animals were randomized for in vivo experiments, but randomization was not performed for all other experiments. Statistical analyses were performed using Student's t-test or two-way ANOVA for most comparisons. Experiments were carried out with three or more replicates unless otherwise stated. Non-parametric Kolmogorov-Smirnov (KS) test was used to evaluate the statistical significance of differential expression between primary prostate cancer and bone metastatic prostate cancer. All experiments were performed in biological triplicates unless otherwise specified. P < 0.05 was considered statistically significant.

Detailed methods are described in Supplementary Information.

## Results

### Identification of specific eRNA modification in bone mPCa

**Figure 1:**
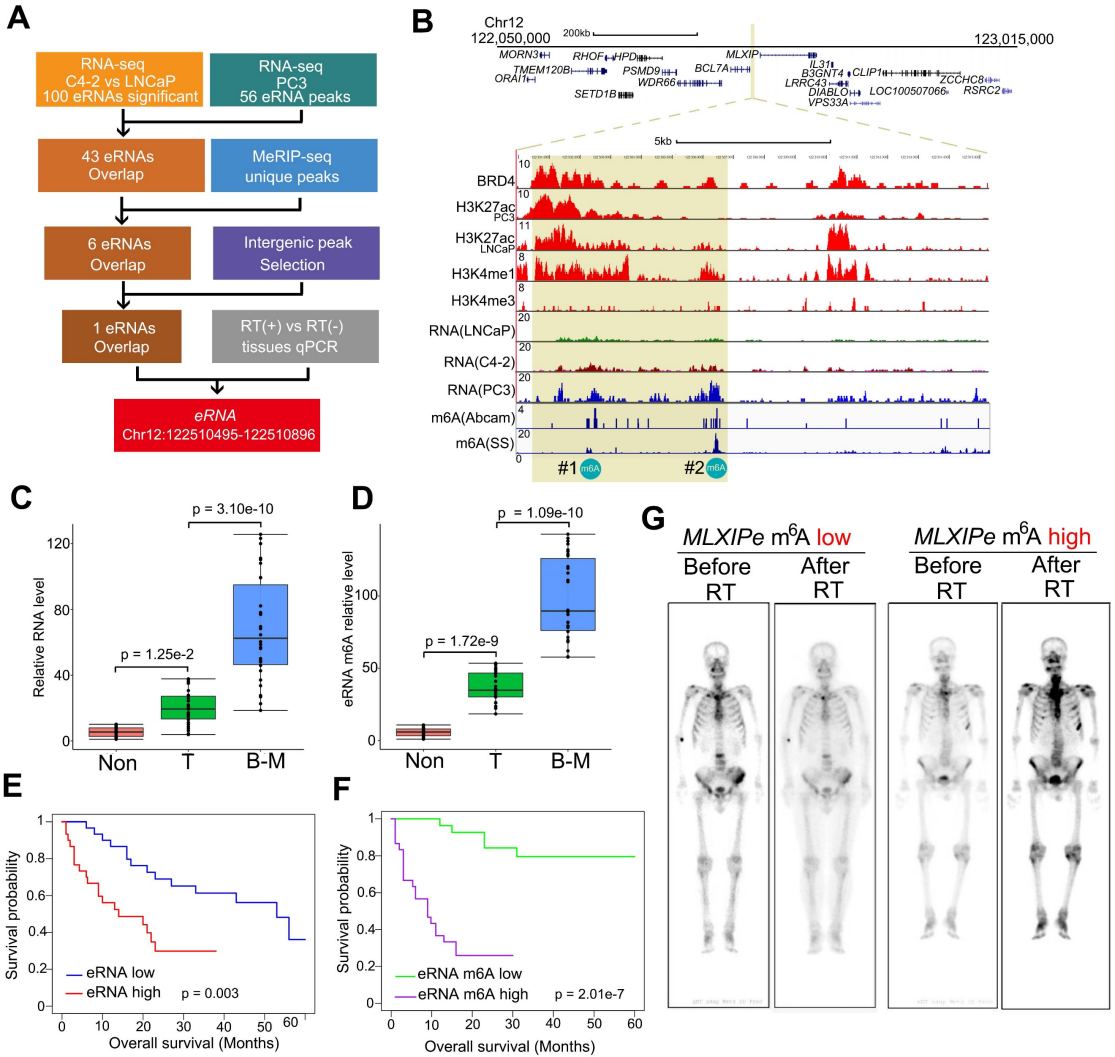
** m^6^A of *MLXIPe* was involved in metastasis of prostate cancer and radiotherapy**. (**A**) Schematic of eRNA filtering analysis. Numbers of RNAs and methods were shown in the boxes. (**B**) Screen shots from UCSC genome browser showing signal profiles of eRNA and mRNA expression in LNCaP. C4-2, PC3; ChIP-seq in LNCaP and PC3 were shown as the references 39. The enhancer regions were highlighted in yellow box. (**C** and **D**) Box and whisker plot showing eRNA expression and m^6^A signals upregulated in bone metastatic prostate cancer tissues. Analysis was of Tianjin Medical University data sets for levels of 12-m^6^A was based on the m^6^A-RIP and RT-PCR. n = 30 each group. *P* values were shown in the figures. (**E** and** F**) Kaplan-Meier survival analysis was of the Tianjin Medical University patient tissues cohort for the relationship between the levels of expression of *MLXIPe* (E) or #2-m^6^A of *MLXIPe* (F) and survival time in bone mPCa tissues. n = 30. *P* values were shown in the figures. (**G**) The PET-scan for metastatic prostate cancer before or after total 30 Gy ^177^Lu-prostate-specific membrane antigen (PSMA)-radioligand therapy-treatment in P-73 (*MLXIPe* m^6^A low) and P-90 (*MLXIPe* m^6^A high) patients.

Compared to primary PCa, more than 30% of advanced prostate cancers were more RT-resistant 7, 8. We analyzed patient survival with or without radiation to investigate the relationship between RT resistance and bone mPCa clinicopathologic characteristics. Consistent with clinical findings from other groups 7, 9, we found that PSMA radioligand therapy prolonged the survival time for patients with primary PCa and lymph node (LN) mPCa, but was limited for patients with bone mPCa in our cohort (Figure S1A). Since enhancers and eRNAs are tissue-specific and function-specific elements, we explored the specific eRNAs in bone mPCa and characterized the bone mPCa-related function of the epitranscriptome. We previously reported stage-related eRNAs in LNCaP (hormone hormone-naïve PCa cell line), C4-2 (CRPC cell line) and PC3 (bone-mPCa cell line) cells using RNA-seq 39, 50. Among the 100 differentially upregulated enhancer transcripts identified by RNA-seq in CRPC cells, we found 43 eRNAs were highly expressed in bone mPCa PC3 cells (Figure 1A and Table S1). Oncogenic activity by genomic enhancers or eRNAs would be the specific targeting therapy in prostate cancer. A recent publication reported that m^6^A in eRNAs was involved in gene transcription in breast cancer cells 40. Thus, we examined whether m^6^A was a specific modification at eRNA in oncogenic bone mPCa. We found 6 eRNAs with m^6^A signals by immunoprecipitating with two different m^6^A antibodies in combination with MeRIP-seq 48. Subsequently, filtering the 6 eRNAs with intergenic peak selection and qPCR verification resulted in a unique m^6^A eRNA upregulated in bone-mPCa cells (Figure 1A-B). A meta-analysis of published ChIP-seq data from LNCaP and PC3 cells identified the region as an authentic enhancer, as evident by the enrichment of enhancer activator (BRD4), enhancer markers (H3K4me1 and H3K27ac) 30, and promoter marker (H3K4me3) (Figure 1B). Based on the naming rules for eRNAs, we labeled this eRNA as *MLXIPe* due to its proximity to *MLXIP* mRNA. Two putative m^6^A sites (#1: TTACA chr12 122502280-122502284; #2: GGACA chr12 122506423-122506427) were identified in *MLXIPe* by two independent m^6^A antibodies (Figure 1B).

We harvested RNA immunoprecipitated by the m^6^A antibody from fresh samples of primary prostate cancer, adjacent normal prostate tissue, and bone metastatic cancer to demonstrate the functional role of m^6^A modifications in a clinicopathological context. Box plot analysis revealed that #2-m^6^A levels of *MLXIPe* were significantly higher in bone-mPCa samples than in primary PCa or adjacent prostate samples in the Tianjin Medical University Urology cohort (Figure 1C-D). However, there was no significant difference in the #1-m^6^A levels of *MLXIPe* between the metastatic prostate cancer, primary prostate cancer, and adjacent prostate tissue samples (Figure S1B). Next, we harvested RNAs from LNCaP, C4-2, PC3, and C4-2B cell lines and found that #2-m^6^A levels of *MLXIPe* were significantly higher in bone metastatic prostate cancer cell lines PC3 and C4-2B (Figure S1C-S1D), but there was no difference in the #1-m^6^A level of *MLXIPe* between all cell lines (Figure S1E). We further determined the survival significance of#2-m^6^A level of *MLXIPe* with clinical characteristics of PCa patients in the bone metastasis cohort. Kaplan-Meier survival analysis of the Tianjin Medical University cohort found that elevated RNA and m^6^A levels of *MLXIPe* were associated with shorter survival in PCa patients with bone metastasis (Figure 1E-F). We also examined the m^6^A levels of *MLXIPe* in patients with PMSA PET. PET analysis showed that in prostate cancer patient 73 (P-73)^MLXIPe-m6Alow^, distant bone metastatic tumor signal disappeared after RT, but stronger signals were detected on both sides of the spine and pelvis in P-90^MLXIPe-m6Ahigh^ (Figure 1G), suggesting RT resistance in MLXIPe-m^6^A^high^ patients. Taken together, *MLXIPe* with m^6^A modification was specific for bone mPCa cells.

### *MLXIPe*-induced RT resistance of mPCa via *PSMD9* upregulation

**Figure 2:**
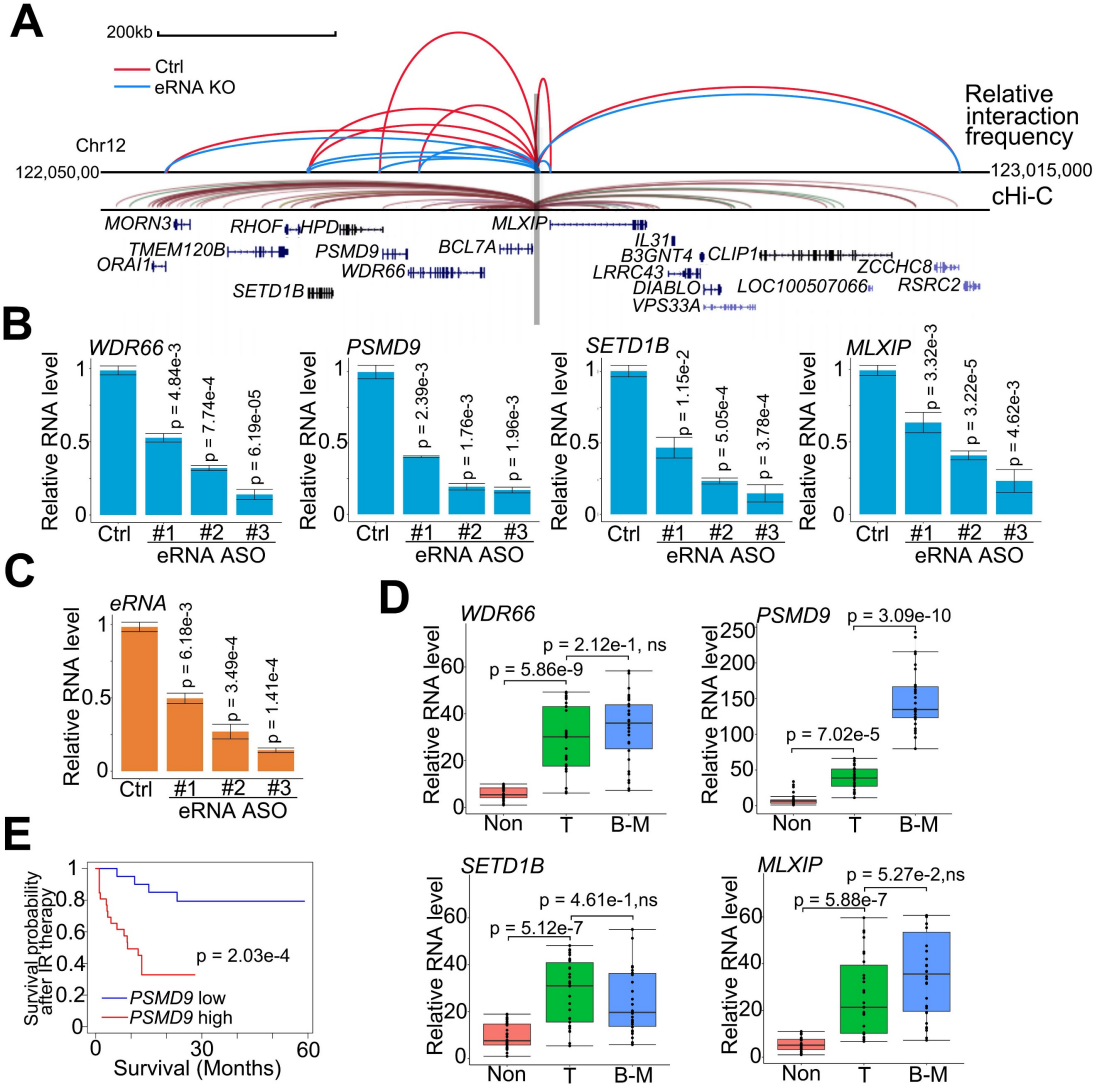
***PSMD9* was the target gene regulated by *MLXIPe* and was involved in radiotherapy resistance.** (**A**) Normalized three-dimensional DNA selection and ligation capture data from *ORAI1* locus to *RSRC2* locus in chromatin 12. Capture 4C data was analyzed from website http://3dgenome.fsm.northwestern.edu. (**B** and **C**) *MLXIPe* and mRNAs RNA expressions were measured by qRT-PCR in P-90 primary cells with three independent ASOs targeting *MLXIPe*. Data presented were means ± standard deviations (SD) of up to three independent replicates. P values were shown in the figures. (**D**) Box and whisker plot showing WDR66, PSMD9, SETD1B and MLXIP expressions elevated in prostate cancer tissues. Analysis was of Tianjin Medical University data cohort for levels of mRNAs were based on the PCR data.n = 30 each group. P values were shown in the figures. (**E**) Kaplan-Meier survival analysis was of the Tianjin Medical University cohort for the relationship between the levels of expression of PSMD9 RNA levels and survival time in bone mPCa tissues after radiotherapy. n = 26. P values were shown in the figures.

eRNAs transfer enhancer activity to one or several neighboring target promoters by increasing enhancer-promoter looping 37, 51. To determine the downstream promoters regulated by *MLXIPe*'s enhancer, we investigated the adjacent promoters using a strategy analogous to chromosome conformation capture (3C) and analyzed published data using the capture Hi-C (cHi-C) technique 52. Enhancer-promoter looping by cHi-C identified 11 mRNAs around *MLXIPe* (Figure 2A). By excluding 4 mRNAs with no expression in prostate cancer cells by RNA-seq data (Figure S2A), we determined the spatial organization of 7 promoters by 3C assay in human bone-mPCa patient-derived xenograft (PDX) primary cell line-90 (P-90). 3C data showed that depletion of *MLXIPe* by antisense oligodeoxynucleotides (ASOs) decreased specific enhancer-promoter interaction between *MLXIPe* and *WDR66* (also called *CFAP251*), *PSMD9*,* SETD1B,* and *MLXIP* loci (Figure 2A and S2B). After identifying the *MLXIPe* target genes by genomic DNA interactions, we investigated whether *MLXIPe* affected their transcription. We knocked down *MLXIPe* by ASOs to verify the eRNA function on the expression of the target. Reverse transcription qPCR and RNA-seq data using three independent ASOs showed that *MLXIPe* downregulated the expression of the four target genes in bone-mPCa primary cell line P-90 and PC3 cells (Figure 2B-C, S2C and Table S2). Interestingly, meta-data showed that the expression of *PSMD9,* but not the other 3 genes, was highly specific in bone marrow (Figure S2D). We also found that *PSMD9* was a specific bone-mPCa-related gene, but *WDR66*, *SETD1B,* and *MLXIP* were oncogenic genes (Figure 2D). PSMD9 is related to 26S proteasome expression and could predict RT benefits in breast cancer 53, 54. Higher levels of *PSMD9* were associated with lower recurrence-free survival after RT (Figure 2E), which was consistent with the association of *PSMD9* with disease-free survival in TCGA data (Figure S2E). Collectively, *PSMD9*, as an *MLXIPe* bone-specific target gene, was correlated with RT resistance and shorter survival in bone mPCa patients.

### *MLXIPe* interacted with KHSRP through m^6^A

**Figure 3:**
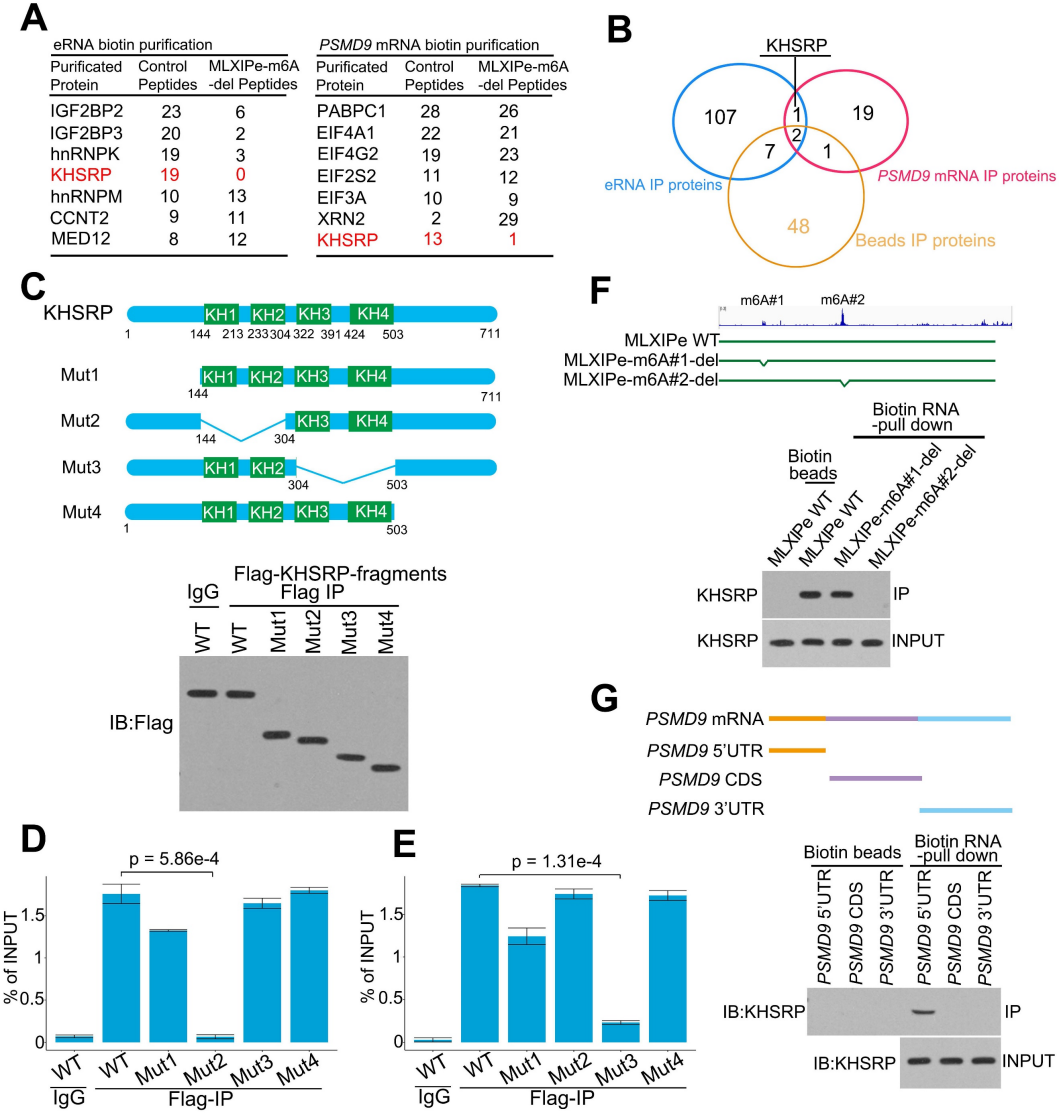
** KHSRP bound to eRNA and mRNA to form a complex.** (**A**) The top hits of *MLXIPe* and* PSMD9* RNA pull-down proteins were identified by TAP-MS. The numbers of peptides were indicated in the list. (**B**) Venn diagram showing that proteins interacting by *MLXIPe* and* PSMD9* RNA pull-down proteins overlapped in P-90 cells. (**C**) Upper, protein diagrams with hnRNP K homology (KH) motif were shown. Lower, western blot of Flag binding at the KHSRP fragments in P-90 cells transfected with Flag-KHSRP-WT, Flag-KHSRP-muts (N-terminal, KH1/2, KH3/4, C-terminal deletion). (**D** and **E**) CLIP-qPCR analysis of KHSRP binding at the *MLXIPe* and* PSMD9* mRNA in P-90 cells with different primers targeting different regions. (**F**) RNA pull-down analysis of KHSRP binding at the *MLXIPe* RNA in P-90 cells transfected with WT, #1-m^6^Am and #2-m^6^A deletion using CRISPR system. (**G**) RNA pull-down analysis of KHSRP binding at the *PSMD9* mRNA in P-90 cells with different probes targeting 5'UTR, CDS and 3'UTR regions.

We explored the molecular mechanism of m^6^A at *MLXIPe* by generating CRISPR control (Ctrl) and *MLXIPe* #2-m^6^A site-deletion (-del) cell lines (Figure S3A) for unbiased tandem affinity purification by biotin-labeled probes targeting *MLXIPe* eRNA or *PSMD9* mRNA. Subsequently, we performed mass spectrometry, showing that *MLXIPe* eRNA bound to multiple proteins, including the looping protein MED12 and KH domain proteins hnRNPK, IGF2BP2, or KHSRP in control (Ctrl) cells but not to KH domain proteins in *MLXIPe* -m^6^A-del cells (Figure 3A and Table S3). Similarly, *PSMD9* mRNA bound to several proteins, including translation protein eIF4A1, eIF4G2, and the KH domain protein KHSRP in Ctrl cells, but not KH domain proteins in eRNA-m^6^A-del cells (Figure 3A and Table S3). KHSRP, an RNA binding protein involved in multiple cancers 55-58, was the common protein between both pull-down groups (Figure 3B). We deleted four domains (N-terminal, KH1/2, KH3/4, or C-terminal-deletion) in the KHSRP protein to confirm the mass spectrometry data (Figure 3C). The cross-linking immunoprecipitation (CLIP)-PCR assay showed that the KH1/2-deletion domain blocked the interaction between the KHSRP protein and *PSMD9* mRNA (Figure 3D), and the interaction between KHSRP and *MLXIPe* was significantly inhibited by KH3/4-deletion domain (Figure 3E).

**Figure 4:**
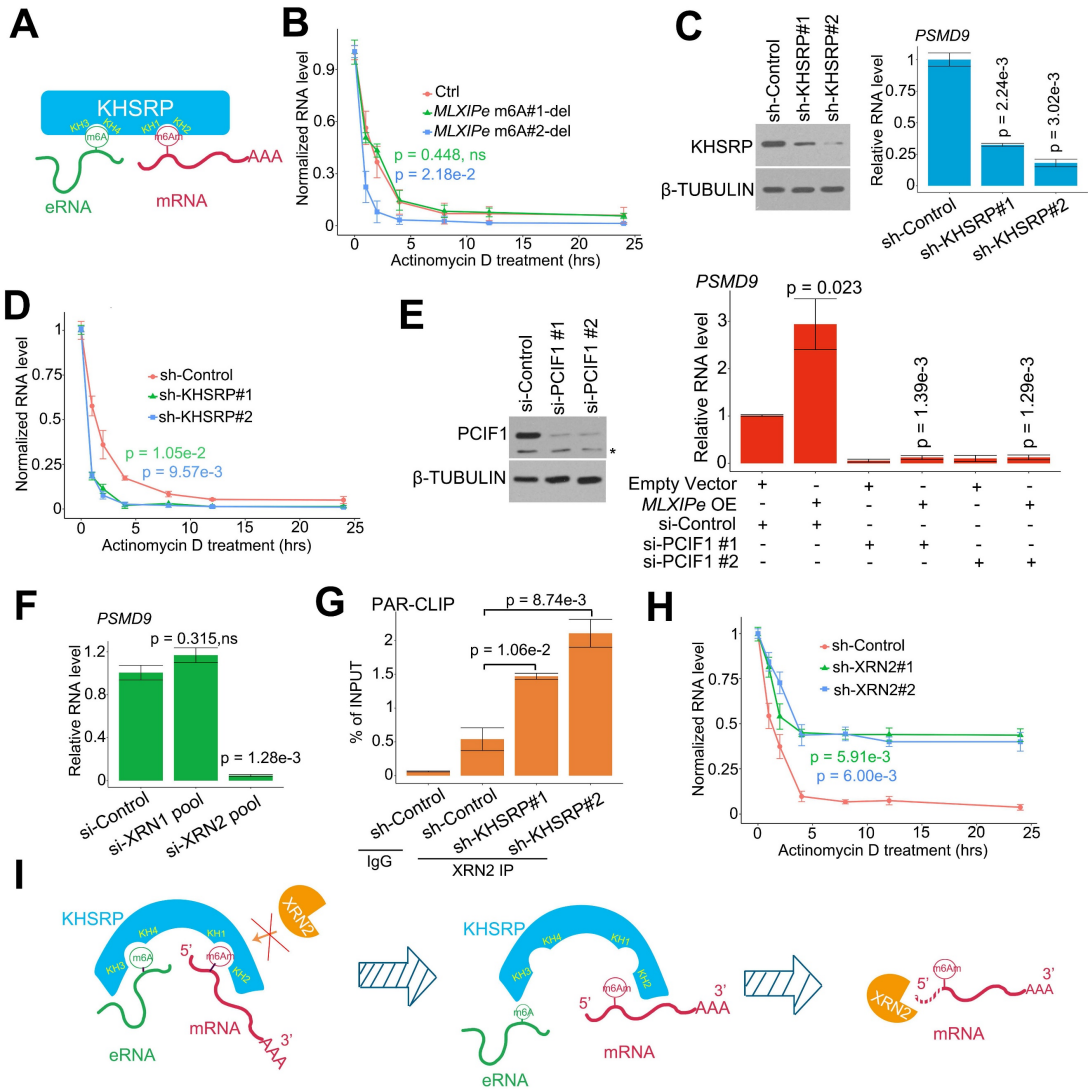
** eRNA-KHSRP-mRNA complex protected *PSMD9* mRNA against from XRN2.** (**A**) A diagram showed eRNA-KHSRP-mRNA complex model. (**B**) A curve-figure showed *PSMD9* mRNA half-life in P-90 cells. The cells were treated with Actinomycin D in time dependent manner. Data presented were means ± standard deviations (SD) of up to three independent replicates. Visible *P* values were shown in the figures. (**C**) Western blot (WB) and qPCR assay showed KHSRP knocking down (KD) in P-90 cells. Cells were transfected with sh-control and sh-KHSRPs, after 48 hrs the cells were harvested for WB or qPCR assay. Means and standard deviations (error bar) were determined from three replicates. *P* values were shown in the figures. (**D**) A curve-figure showed *PSMD9* mRNA half-life in P-90 cells. The cells were transfected with sh-control and sh-KHSRPs and treated with Actinomycin D in time dependent manner. Data presented were means ± standard deviations (SD) of up to three independent replicates. P values were shown in the figures. (**E**) Western blot (WB) and qPCR assay showed PCIF1 knocking down in P-90 cells. Cells were transfected with si-control or si-PCIF1s and empty vector or *MLXIPe* RNA, after 48 hrs the cells were harvested for WB or qPCR assay. Means and standard deviations (error bar) were determined from three replicates. Visible *P* values were shown in the figures. (**F**) qPCR assay XRN2 knocking down (KD) in P-90 cells. Cells were transfected with si-control and si-XRN2s, after 48 hrs the cells were harvested for qPCR assay. Data presented were means ± standard deviations (SD) of up to three independent replicates. P values were shown in the figures. (**G**) CLIP-qPCR analysis of XRN2 binding at the *PSMD9* mRNA in P-90 cells transfected with sh-control and sh-KHSRPs. (**H**) A curve-figure showed *PSMD9* mRNA half-life in P-90 cells. The cells were transfected with si-control and si-XRN2s and treated with Actinomycin D in time dependent manner. Data presented were means ± standard deviations (SD) of up to three independent replicates. P values were shown in the figures. (**I**) A diagram showed how eRNA-KHSRP-mRNA complex protected mRNA against from degradation by XRN2.

Next, we examined the effect of m^6^A modification on the interaction between KHSRP and *MLXIPe* and observed that #2-m^6^A site deletion blocked *MLXIPe* association with KHSRP, whereas #1-m^6^A site deletion failed to affect the interaction (Figure 3F and S3A). Furthermore, KHSRP bound to 5'UTR of *PSMD9* mRNA but not to CDS and 3'UTR regions (Figure 3G). 5'UTR of mRNAs is methylated with *N*^6^,2'-*O*-dimethyladenosine (m^6^Am), which is also recognized by the m^6^A antibody 19. It was important to characterize whether KHSRP bound to m^6^Am or m^6^A at 5'UTR of *PSMD9* mRNA. For this, we analyzed the published m^6^Am-seq and performed epitranscriptome analysis to distinguish m^6^Am and m^6^A in cells through L-ascorbic acid changing Fe(II)/2 related affinity activation 49. Thus, m^6^Am signals could be reduced by FTO treatment, but m^6^A signals were not changed 49, such as on the *RBM48* locus (Figure S3B). The high-throughput sequencing data showed m^6^Am modification at the 5'UTR of *PSMD9* and *MLXIP* mRNAs, suggesting that KHSRP bound to m^6^Am using its KH1/2 domain (Figure S3B). m^6^Am-seq showed an m^6^Am at the #1 site of *MLXIPe* (Figure S3B), explaining the reason the #1 m^6^Am-site of *MLXIPe* failed to interact with m^6^A reader IGF2BP2 or IGF2BP3 (Figure 3A and S3B). To further demonstrate that m^6^Am is necessary for KHSRP-*PSMD9* interaction, we generated m^6^Am-deletion *PSMD9* mRNA 5'UTR plasmids by QuickChange II Site Directed-Mutagenesis-kit. We found that m^6^Am deletion of *PSMD9* mRNA 5'UTR fragment failed to bind KHSRP in PC3 cells (Figure S3C), suggesting that m^6^Am was necessary for the interaction between KHSRP and *PSMD9* mRNA. The simultaneous interaction of KHSRP with eRNA and mRNA was examined by testing eRNA using *PSMD9* pull-down assay and *PSMD9* mRNA using eRNA pull-down assay with or without knocking down KHSRP. The data showed that KHSRP interacted with both eRNA and mRNA simultaneously (Figure S3D). Thus, KHSRP was an m^6^A and m^6^Am double reader that interacted with m^6^Am using the KH1/2 domain and with m^6^A using the KH3/4 domain (Figure 4A).

### KHSRP stabilized mRNA by connecting it with eRNA

It has been reported that m^6^Am is involved in mRNA stability 19, 25, 28, 59. We examined the half-life of RNA in P-90 cells to confirm the relevance m^6^Am in mRNA stability. The RNA half-life data indicated that #2 m^6^A deletion increased *PSMD9* mRNA degradation, but #1-m^6^Am site deletion failed to complete it in P-90 and PC3 cells (Figure 4B and S4A). We analyzed the relationship among *MLXIPe*, *PSMD9* mRNA and m^6^A levels in the clinical cohort. The data showed that *MLXIPe* m^6^A levels were significantly correlated with* MLXIPe* RNA and *PSMD9* mRNA levels, suggesting that *MLXIPe* m^6^A modifications upregulated the stabilization of *MLXIPe* and *PSMD9* mRNAs (Figure S4B). Meanwhile, KHSRP depletion with two independent shRNAs significantly downregulated *PSMD9* RNA levels and enhanced *PSMD9* mRNA degradation (Figures 4C-D). The m^6^Am writer is PCIF1 as reported 60. PCIF1-KD impaired the *PSMD9* RNA levels elevated by* MLXIPe* (Figure 4E). Importantly, RNA-seq data showed that elevated *MLXIPe* increased the 5'UTR signals in PC3 cells, whereas decreased *MLXIPe* exhibited lower 5'UTR signals in LNCaP cells (Figure S4C). The qPCR assay with paired primers targeting 5'UTR, CDS, and 3'UTR showed that high *MLXIPe* enhanced the ratios of 5'UTR/CDS and 5'UTR/3'UTR at *PSMD9* and *MLXIP* loci (Figure S4C), suggesting that the degradation of *PSMD9* mRNA might be from 5' to 3'. XRN1 and XRN2 are highly processive 5' > 3' exoribonucleases 61. The Knockdown assay demonstrated that XRN2, but not XRN1, was the 5' > 3' exoribonuclease responsible for *PSMD9* mRNA degradation. (Figure 4F). Depletion of KHSRP increased *PSMD9* mRNA binding to XRN2 (Figure 4G). Furthermore, knocking down of XRN2 upregulated the stability of *PSMD9* mRNA (Figure 4H and S4D). These data suggested that *MLXIPe-*KHSRP*-PSMD9* blocked the XRN2-induced degradation of *PSMD9* mRNA in bone mPCa cells (Figure 4I).

### *MLXIPe* elevated levels of DNA repair proteins through m^6^A in prostate cancer cells

**Figure 5:**
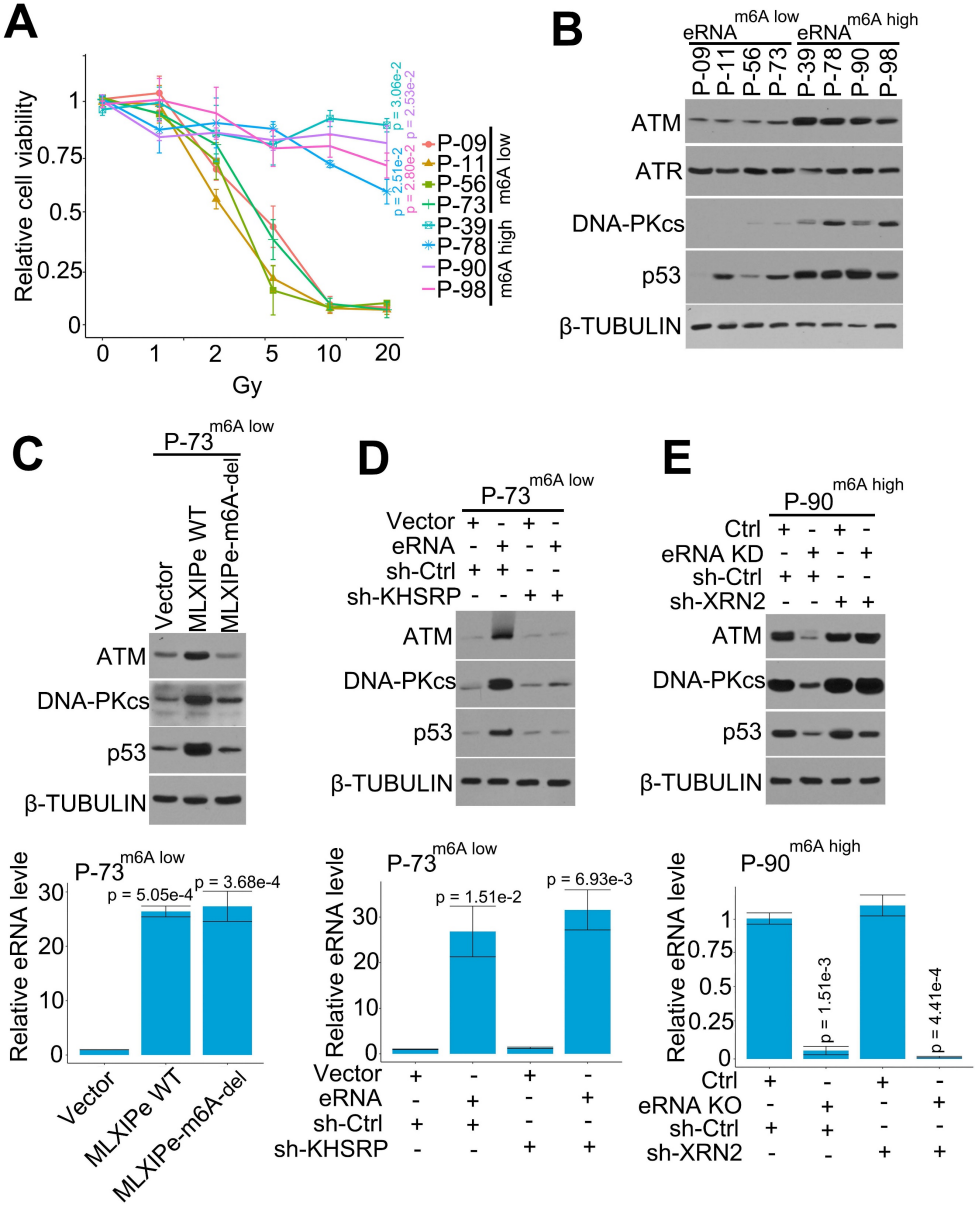
***MLXIPe* elevated key proteins of DNA repair pathway.** (**A**) Relative primary cell viabilities were completed by MTS assay. Bone-mPCa primary cells were radiated in a dose dependent manner, after radiation 72 hrs cells were treated with MTS for 2 hrs and measured. Data presented were means ± standard deviations (SD) of up to three independent replicates. P values were shown in the figures. (**B**) Expression of DNA repair related proteins were measured by western blot in *MLXIPe* m^6^A low and *MLXIPe* m^6^A high primary bone-mPCa cells. (**C**) Western blot and qPCR assay showed eRNA m^6^A knocking out (KO) in *MLXIPe* m^6^A^low^ P-73 cells. Cells were infected with empty vector, *MLXIPe* WT and *MLXIPe* m^6^A-del, after 48 hrs the cells were harvested for western blot or qPCR assay. Data presented were means ± standard deviations (SD) of up to three independent replicates. P values were shown in the figures. (**D**) Western blot and qPCR assay showed eRNA m^6^A and KHSRP knocking down in *MLXIPe* m^6^A^low^ P-73 cells. Cells were infected with empty vector or *MLXIPe* WT accompanying sh-control or sh-KHSRP, after 48 hrs the cells were harvested for western blot or qPCR assay. Data presented were means ± standard deviations (SD) of up to three independent replicates. *P* values were shown in the figures. (**E**) Western blot and qPCR assay showed eRNA knocking-down (KD) by ASO-mix and XRN2 knocking-down by shRNAs in *MLXIPe* m^6^A^high^ P-90 cells. Cells were infected with CRISPR empty vector or CRISPR targeting* MLXIPe* m^6^A site accompanying sh-control or sh-XRN2, after 48 hrs the cells were harvested for western blot or qPCR assay. Data presented were means ± standard deviations (SD) of up to three independent replicates. P values were shown in the figures.

PSMD9 is an RT predicting marker related to RT resistance in breast cancer cells 53, 54. In this study, we found that *MLXIPe* mediated the *PSMD9* mRNA stability and upregulated its protein level via eRNA m^6^A (Figure 4). We selected four *MLXIPe* m^6^A^high^ and *MLXIP* m^6^A^low^ primary bone-mPCa PDX cells from our PDX cohort to determine whether *MLXIPe* m^6^A was the specific RT resistance marker in bone-mPCa. Different dose radiation curve data indicated RT resistance in *MLXIPe* m^6^A^high^ cells; however, *MLXIPe* m^6^A^low^ cells were killed in a dose-dependent manner by radiation (Figure 5A). PSMD9 belongs to 26S proteasome, a multi-catalytic proteinase complex for protein degradation 54. Western blotting showed that ATM, DNA-PKcs, and p53 protein levels were upregulated in *MLXIPe* m^6^A^high^ cells, but the ATR expression was not affected (Figure 5B). We determined whether the functional role of MLXIPe m^6^A in mPCa RT resistance was through PSMD9, by overexpressing PSMD9 and/or knocking down KHSRP in mPCa primary cells, and performing MTS cell survival assay and protein expression by Western blotting. The data showed that PSMD9 depletion impaired cancer cell survival after RT (Figure S5A). Furthermore, we found that the knocking down of KHSRP downregulated ATM, DNA-PKcs, and p53 expression after RT. On the contrary, overexpression of PSMD9 rescued ATM, DNA-PKcs, and p53 protein expression in P-73 and P-90 cells following RT (Figure S5B).

We deleted m^6^A in *MLXIPe* to further confirm m^6^A function in the DNA repair pathway*.* The expression data showed that *MLXIPe* WT but not *MLXIPe-*m^6^A deletion elevated ATM, DNA-PKcs, and p53 protein levels (Figure 5C). Also, *MLXIPe* WT and *MLXIPe-*m^6^A-deletion elevated DNA repair and decreased the number of γ-H2AX foci at 8 h after IR (Figure S5C). The function of the eRNA-KHSRP-mRNA complex in the DNA repair pathway was verified in *MLXIPe* m^6^A^low^ P-73 cells and *MLXIPe* m^6^A^high^ P-90 cells. The data showed that KHSRP-depletion inhibited the upregulation of ATM, DNA-PKcs, and p53 proteins by *MLXIPe* and impaired the ratio of γ-H2AX foci (8h/1h after IR) in* MLXIPe* m^6^A^low^ P-73 cells (Figure 5D and S5D). Meanwhile, XRN2 knock-down rescued ATM, DNA-PKcs, and p53 protein levels and enhanced the ratio of γ-H2AX foci (8h/1h after IR) in *MLXIPe* m^6^A-deleted cells (Figure 5E and S5E). These data indicated that *MLXIPe* elevated ATM, DNA-PKcs, and p53 protein levels through the m^6^A and KHSRP complex in bone-mPCa cells.

### *MLXIPe* enhanced prostate PDX RT resistance through m^6^A and KHSRP

**Figure 6:**
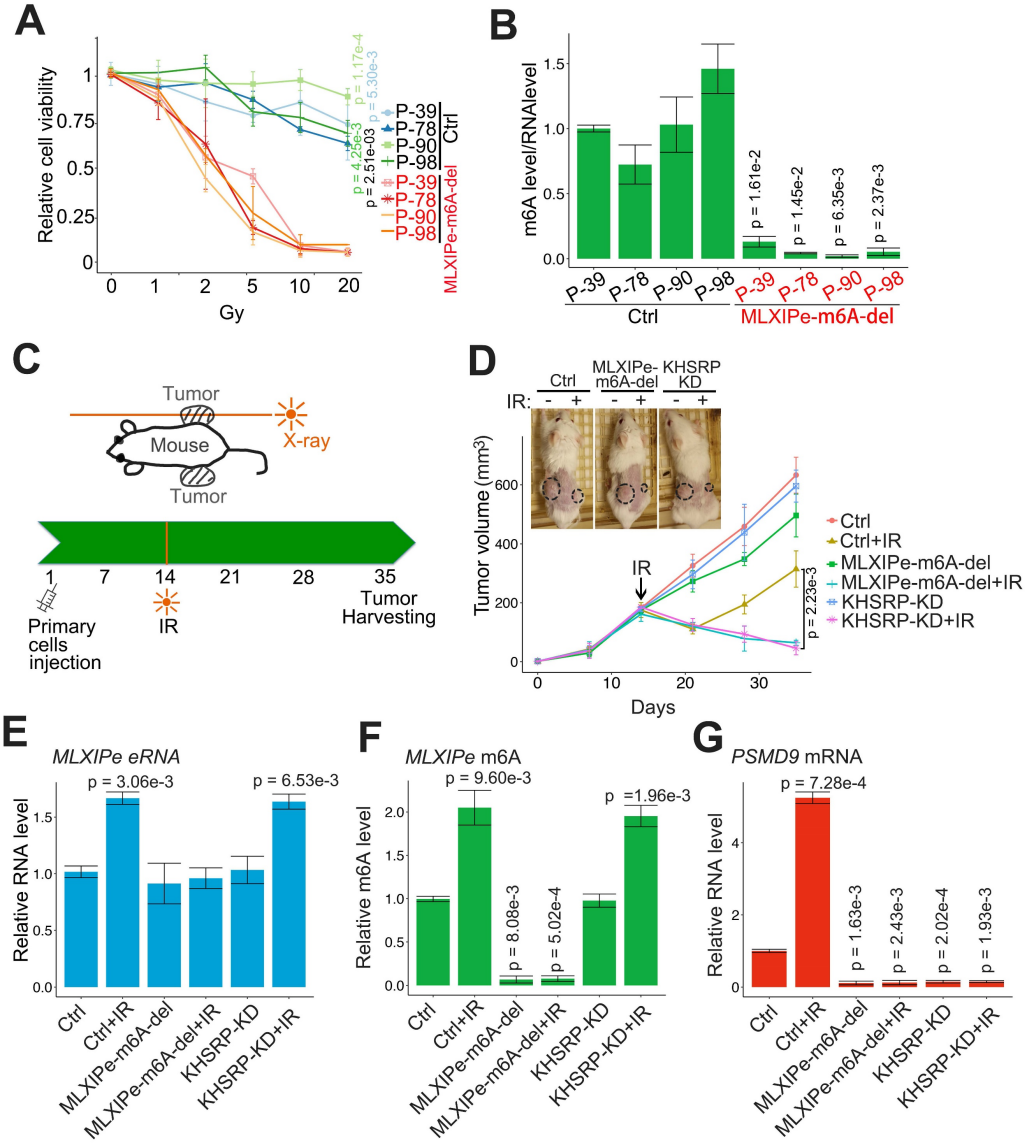
***MLXIPe* induced radiotherapy resistance in vivo.** (**A** and **B**) Relative primary cell viabilities were completed by MTS assay. Bone-mPCa primary cells infected with CRISPR empty vector or CRISPR targeting* MLXIPe* m^6^A site were radiated in a dose dependent manner, after radiation 72 hrs cells were treated with MTS for 2 hrs and measured. The m^6^A status were measured by m^6^A-RIP. Data presented were means ± standard deviations (SD) of up to three independent replicates. P values were shown in the figures. (**C** and **D**) P-90 cells (3x10^6^) transfected with lenti-virus shRNAs were injected into NSG mice flanks (n = 6 each group). The one-side xenografts of mice were subjected to X-ray radiation in 14 days. The tumor growth was observed every 7 days, and the data were shown in the bottom panel. *P* values were shown in the figures. Representative images out of 6 different mice were displayed in 35 day and then were harvest for RNAs and m^6^A measurements. (**E**) *MLXIPe* eRNA expressions were measured by qPCR in P-90 xenograft tissues. Data presented were means ± standard deviations (SD) of up to three independent replicates. P values were shown in the figures. (**F**) Effect of m^6^A of *MLXIPe* on growth of bone-mPCa xenografts. P-90 cells were injected into NSG mice flanks (n = 6 each group). After 35 days the xenografts were harvested for m^6^A-RIP. Data shown as means ± SD (n = 6). Statistical significance was determined by two-tail Student's *t*-test. *P* values were displayed in the figures. (**G**) *PSMD9* mRNA expressions were measured by qPCR in P-90 xenograft tissues. Data presented were means ± standard deviations (SD) of up to three independent replicates. P values were shown in the figures.

We tested the m^6^A function of *MLXIPe* in PDX cells by editing the m^6^A site in four *MLXIPe* m^6^A^high^ PDX cells. The data showed that the RT resistance was impaired and radiosensitivity was recovered in *MLXIPe* m^6^A sites-deleted (-del) mPCa cells (Figures 6A-B). Next, we determined the m^6^A function of *MLXIPe* in vivo in the xenograft model and examined the effect of m^6^A regulation of *MLXIPe* on prostate xenograft growth and radio resistance. When *MLXIPe-*m^6^A-del cells injected into the mice flank, the data showed that *MLXIPe-*m^6^A knock-out slightly decreased the growth of xenografts, whereas KHSRP-KD transfection did not affect xenograft growth (Figure 6C-D). Significantly, *MLXIPe-*m^6^A-del and KHSRP-KD groups recovered radiosensitivity after RT in 36 days, but not the Ctrl group (Figure 6D). The qPCR data showed that *PSMD9* mRNA was associated with the RT resistance of xenografts (Figure 6E-G). Thus, our data indicated that m^6^A of *MLXIPe* or KHSRP elevated the mPCa RT resistance in the PDX model.

## Discussion

Metastatic prostate cancer is the principal cause of mortality in men. Bone is the most common target organ for prostate cancer metastasis in an overwhelming 80 % of patients with a 5-year survival rate of merely 3% 1, 3. Androgen deprivation therapy is one of the main treatments for advanced prostate cancer, however, CRPC is a challenge for prostate cancer treatment by hormone therapy or drugs, especially for metastatic CRPC 62, 63. RT is another critical component of cancer management in over 50% of cancer patients 9, 64. Sometimes RT is the first treatment for cancers that have metastasized outside the prostate gland and into nearby tissues 7. Furthermore, ^177^Lu- PSMA radioligand therapy (RLT) is a novel therapeutic option in patients with metastatic CRPC. RT or hormone therapy was considered an excellent independent therapy. However, combination therapy with RT exhibited an equal overall survival of metastatic CRPC (mCRPC) in recent clinical trials 8, 46, 64, suggesting that mCRPC is not only resistant to hormone therapy but also has the feature of being RT resistant. Therefore, we hypothesized that there is a common specific oncogenic factor in CRPC and mPCa.

In this study, we filtered the m^6^A eRNA in a hormone-naïve PCa cell line LNCaP, CRPC cell line C4-2, and bone-mPCa cell line PC3 by RNA-seq, MeRIP-seq, intergenic selection, and RT-qPCR, and identified a new eRNA *MLXIPe* with m^6^A as the candidate (Figure 1). We demonstrated four target genes of *MLXIPe,* including* WDR66, SETD1B, PSMD9,* and* MLXIP* (Figure 2). Among these,* WDR66, SETD1B,* and* MLXIP* were novel markers for cancer metastasis and involved in epithelial-mesenchymal transition (EMT) 65-67. However, these three genes were not bone-specific (Figure 2 and S2). Importantly, *PSMD9,* the expression of was bone-specific, was involved in the RT resistance of bone mPCa, indicating that the *MLXIPe-PSMD9* axis might be the key pathway mediating bone-mPCa RT resistance.

Despite the discovery of enhancers regulating gene transcription over 40 years, the enhancer-mediated post-transcription is not entirely understood. eRNA was demonstrated as the critical factor in the mechanism of enhancers regulating genes' expression 31, 37, 51. Recently published finding reported that m^6^A at eRNA was involved into breast cancer oncogenesis 40. It supplies two scientific questions whether m^6^A is at the disease-specific-expressed RNA (such as eRNA) in specific cancer cells and whether it is the functional modification in these cancer cells. To ask both questions, we detected the m^6^A status at eRNA in the prostate cancer. Here, unlike m^6^A abundant modification at mRNA, we found about 6% eRNA with m^6^A modification in prostate cancer cells, consistent with published data of about 18% eRNA marked with m^6^A in breast cancer cells 40. Unlike the eRNA function in looping and transcription activation, we found a novel function of eRNA that protected XRN2 to maintain the stability of target mRNA at the post-transcriptional level. As shown in RNA-seq data (Figure 4 and S4), *MLXIPe*^low^ cells exhibited a low ratio of 5'UTR/CDS and 5'UTR/3'UTR, suggesting that *MLXIPe* was involved in the stability of 5' end of mRNAs. KHSRP binding to *PSMD9* mRNA 5'UTR significantly blocked the interaction of XRN2 with *PSMD9* mRNA (Figure 4). These data provided new insight into eRNA post-transcriptional regulation and protection of the nascent mRNA stability from its 5' end.

m^6^A in mammals was identified as a highly abundant modification of mRNA among more than 160 chemical modifications 13-15. Besides m^6^A, there is another reversible modification in eukaryotes known as m^6^Am. Unlike the m^6^A mainly located at 3'UTR, m^6^Am is precisely located at the first transcribed nucleotide and hence adjacent to the mRNA cap 19. Recently, a limited number of writers, erasers, and readers of m^6^A were reported by many groups 18, 26, 28. m^6^Am has been shown to be catalyzed by PCIF1, an interacting protein with RNA polymerase II 19, 49. The only eraser of m^6^Am is FTO, which is also the same enzyme as m^6^A 49, 68. However, the reader of m^6^Am remained unclear.

Using the published data of m^6^Am-seq 49, we distinguished m^6^Am and m^6^A in eRNAs and mRNAs (Figure S3). Consistent with our mass spec data in Figure 3A, #2 site m^6^A of *MLXIPe* bound to KHSRP, IGF2BP2, and IGF2BP3 of which IGF2BP2 and IGF2BP3 are m^6^A readers^69^, ^70^, but #1 site of the putative m^6^A of *MLXIPe* failed to bind because it was an m^6^Am at eRNA as determined by m^6^Am-seq. Furthermore, MeRIP-seq (Figure 1B) showed that #1-site m^6^Am level was about 5 times lower than #2-site methylation level, suggesting that #1-site was a weaker eRNA methylation site than m^6^Am at the mRNA. Also, the RNA pull-down data displayed in Figure 3G showed that the KH1/2 domain of KHSRP bound to 5'UTR of *PSMD9* mRNA, demonstrating that the KH1/2 domain of KHSRP was a potential reader of m^6^Am. Significantly, we found an m^6^Am and m^6^A double-recognized protein with different KH domains. The m^6^Am interaction mechanism was assisted by *MLXIPe* binding (Figure 3A). When eRNA-m^6^A was disassociated with the KHSRP KH3/4 domain, KHSRP did not bind to m^6^Am at *PSMD9* mRNA (Figure 3A), suggesting that the eRNA-KHSRP interaction may change the KH1/2 structure and facilitate the interaction between KHSRP and m^6^Am at the *PSMD9* mRNA. Functionally, the new complex of eRNA-KHSRP-mRNA protected the 5' end of *PSMD9* mRNA from degradation by XRN2 in bone-mPCa cells. These data indicated that the new m^6^Am read complex played an RT-resistant role through PSMD9 in bone-mPCa cells.

In summary, our data provided new insights into mPCa RT resistance, eRNA modification, and the mechanism of the potential m^6^Am reader. We detected a bone-specific eRNA with m^6^A that facilitated the RT resistance function in bone metastatic prostate PDXs. KHSRP recognized and interacted with m^6^A at the eRNA and m^6^Am on 5'-UTR of mRNA to block RNA degradation from 5'-UTR by XRN2. The findings indicated that a bone-specific eRNA-m^6^A is vital in regulating mPCa growth and RT resistance and might be a novel target for cancer therapy (Figure 6). Our study highlights *MLXIPe* as a potential predictive biomarker and therapeutic target for bone-mPCa.

### Availability of data and materials

All data that support the findings of this study are available from the corresponding authors upon reasonable request.

## Supplementary Material

Supplementary figures and methods.

Supplementary table 1.

Supplementary table 2.

Supplementary table 3.

Supplementary table 4.

## Abbreviations

CRPC: castration-resistant prostate cancer
CLIP: Cross-linking immunoprecipitation
eRNAs: Enhancer RNAs
mPCa: Metastatic prostate cancer
m^6^Am: *N*^6^,2'-*O*-dimethyladenosine
m^6^A: N^6^-methyladenosine
PCR: Polymerase chain reaction
qPCR: Quantitative real-time PCR
RT: Radiotherapy
RIP: RNA immunoprecipitation
5′ UTR: 5′-untranslated region
3′ UTR: 3′-untranslated region
