# Supplementary material for: Enhancer RNA promotes resistance to radiotherapy in bone-metastatic prostate cancer by m6A modification
Source: Theranostics. 2023 Jan 1;13(2):596–610. doi: 10.7150/thno.78687 (PMC9830431; doi:10.7150/thno.78687)
Supplement: Supplementary file 1 — Supplementary figures and methods. [file thnov13p0596s1.pdf]

Supplementary Information

Supplementary figure legends

Figure S1. m6A of *MLXIPe* was a negative prognostic factor for patients.
(A) Kaplan-Meier survival analysis was of the Tianjin Medical University cohort for the relationship between the patients with or without ^{177}Lu-prostate-specific membrane antigen (PSMA)-radioligand therapy in PCa, Lymph node mPCa or bone mPCa. *P* values were shown in the figures.
(B and C) Box and whisker plot showing eRNA expression and m^{6}A signals upregulated in bone metastatic prostate cancer tissues. Analysis was of Tianjin Medical University data sets for levels of #2-m^{6}A and *MLXIPe* RNA were based on the m^{6}A-RIP and RT-PCR. n=30 each group. *P* values were shown in the figures.
(D and E) #2-m^{6}A and *MLXIPe* RNA expressions were measured by m^{6}A-RIP and qPCR in LNCaP, C4-2, C-2B, PC3 cells. Data presented were means ± standard deviations (SD) of up to three independent replicates. P values were shown in the figures.

Figure S2. Enhancer-promoter interaction of *MLXIPe* and its target genes.
(A) Screen shots from UCSC genome browser showing signal profiles of eRNA and mRNA expression in LNCaP. C4-2, PC3.
(B) The diagram showing that chromosome conformation capture (3C) assay with EcoRI digesting in *WDR66* locus, BamHI digesting in *PSMD9* locus, NcoI digesting in *SETD1B* locus, and PstI digesting in *MLXIP* locus. Yellow box showed the digestion site with primer. Red arrows showed forward primers, yellow arrows showed reverse primers.
(C) *MLXIPe* and mRNAs RNA expressions were measured by qRT-PCR in PC3 cells with three independent ASOs targeting *MLXIPe*. Data presented were means ± standard deviations (SD) of up to three independent replicates. P values were shown in the figures.
(D) Tissue specific expression data were from The Human Protein Atlas. The specific high expression was shown in red box.
(E) Kaplan-Meier survival analysis was of the TCGA data sets for the relationship between the levels of expression of *PSMD9* in prostate cancer tissues. n=123. *P* values were shown in the figures.

Figure S3. m^{6}A and m^{6}Am determination by high though sequences.
(A) Genomic DNAs were harvested from parental and CRISPR stable cells. PCRs were performed using the primers shown in the diagram.
(B) Screen shots from UCSC genome browser showing m^{6}A signal profiles of eRNA and mRNA expression in 293T cells with or without FTO. The m^{6}A regions were highlighted in orange box, The m^{6}Am regions were highlighted in green box.

(C) *PSMD9* mRNA levels were measured by CLIP with KHSRP antibody and qPCR in P-90 cells. Data presented were means ± standard deviations (SD) of up to three independent replicates. P values were shown in the figures.
(D) *PSMD9* mRNA (Left) and *MLXIPe* RNA (Right) levels were measured by RNA pull-down and qPCR in P-90 cells. Data presented were means ± standard deviations (SD) of up to three independent replicates. P values were shown in the figures.

Figure S4. mRNAs were degraded by XRN2 from 5'-end.
(A) A curve-figure showed *PSMD9* mRNA half-life in PC3 cells. The cells were treated with Actinomycin D in time dependent manner. Data presented were means ± standard deviations (SD) of up to three independent replicates. Visible *P* values were shown in the figures.
(B) *MLXIPe*, m^6A levels and *PSMD9* mRNAs RNA expressions were measured by qRT-PCR in 30 tissue samples. R value and P values were shown in the figures.
(C) Upper, screen shots from UCSC genome browser showing signal profiles of eRNA and mRNA expression in LNCaP. C4-2, PC3; ChIP-seq were shown as the markers of enhancer and promoter. The 5'UTR, exon2 and 3'UTR regions were highlighted in yellow box. Lower, Ratios of 5'UTR/Exon2 or 5'UTR/3'UTR were completed by qPCR. Means and standard deviations (error bar) were determined from 3 replicates. *P* values were shown in the figures.
(D) Western blot assay showed XRN2 knocking down (KD) in P-90 cells. Cells were transfected with sh-control and sh-XRN2s, after 48 hrs the cells were harvested for WB assay.

Figure S5. *MLXIPe* elevated DNA repair.
(A) Relative primary cell viabilities were completed by MTS assay. Bone mPCa primary cells with or without PSMD9 overexpression and/or KHSRP depletion were radiated in a dose dependent manner, after radiation 24 hrs cells were treated with MTS for 2 hrs and measured. Data presented were means ± standard deviations (SD) of up to three independent replicates. P values were shown in the figures.
(B) Expression of DNA repair related proteins were measured by western blot in primary bone mPCa cells with or without PSMD9 overexpression and/or KHSRP depletion.
(C and D) P-73 primary cells infected with EV or eRNA or eRNA-m6A-deletion (KO) or KHSRP-knocking down were treated with 4Gy IR. At each time point after IR, the cells were harvested for IF with antibodies for γ-H2AX. The average foci number in each cell were quantified. Data are presented as means ± SD of more than 30 cells from six biological replicates. *P* values were shown in the figures.
(E) P-90 primary cells infected with EV or eRNA or XRN2-knocking down were treated with 4Gy IR. At each time point after IR, the cells were harvested for IF with antibodies for γ-H2AX. The average foci number in each cell were quantified. Data are presented as means ± SD of more than 30 cells from six biological replicates. *P* values were shown in the figures.

Supplementary Methods

Human prostate cancer specimens and RNA isolation from human tissues

Formalin-fixed paraffin-embedded (FFPE) hormone-naïve primary prostate cancer and bone metastatic tissues were randomly selected from the Tianjin Medical University Tissue Registry. Hormone-naïve patients with biopsy-proven prostate cancer have been treated at Tianjin Medical University by radical retropubic prostatectomy between January 1995 and December 2018 with radiotherapy. The study was approved by the Tianjin Medical University Institutional Review Board. FFPE tissues were collected and total RNAs were isolated using a RecoverAll Total Nucleic Acid Isolation Kit (Life Technologies). Isolation of RNAs from frozen human prostate cancer tissues was performed as described previously (Zhao *et al*, 2019; Zhao *et al*, 2016).

RNA isolation from cultured cells, reverse transcription PCR (RT-PCR) and real-time PCR

Total RNA was isolated from cultured cells using TRIzol reagent (Invitrogen) or the RNeasy Plus Mini Kit (Qiagen) according to the manufacturer's instructions. First-strand cDNA was synthesized with the PrimeScript Reverse Transcriptase Kit (TaKaRa Bio). Reverse transcription and real-time PCR were performed as described previously (Zhao *et al.*, 2019). The PCR primers for AR new target genes are listed in Table S3.

Patient-derived xenograft (PDX)

PDXs were propagated in BALB/c nu/nu mice and AI xenografts were propagated in SCID mice. Mice were housed in the Tianjin Medical University pathogen-free rodent facility. All procedures were approved by the Tianjin Medical University Institutional Animal Care and Use Committee (Wen *et al*, 2020).

Positron emission tomography (PET) Image Acquisition, Reconstruction and Analysis

The ligand was ^{68}Ga-PSMA-11 (Glu-NH-CO-NH-Lys-(Ahx)-[^{68}Ga(HBED-CC)]). For therapy, organs at risk for a critical radiation dose are the kidneys (0.5–0.8 Gy/GBq; conservative dose limit, 23 Gy (31)) and the lacrimal and salivary glands (0.6–1.4 Gy/GBq; dose limit not clearly established (31)) (8,10–12,17,32). The radiation dose to the bone marrow (<0.05 Gy/GBq (8,11,12,17)) and the liver and spleen (each < 0.5 Gy/GBq (12,17,32)) is considerably below critical thresholds. The median injected activity was 198 MBq (5.3 mCi) (range, 107.3–233.1 MBq [2.8–6.4 mCi]). The median tracer uptake period was 1 h (range, 46–110 min). The images were acquired using a PET/CT scanner (Infinia Hawkeye4; GE) (Calais *et al*, 2018). The PET image contained a whole-body scan (pelvis to vertex, 4 min/bed position depending on the patient weight), 1 dedicated pelvic scan after voiding (same acquisition time/bed position time as used for the whole body), and 1 dedicated scan of the lower extremities (pelvis to toes, 1 min/bed position for a total of 10 min). The PET images were reconstructed using random-event, attenuation, dead-time and scatter corrections. PET images were reconstructed with an iterative algorithm (ordered-subset expectation maximization) in an axial 168 × 168 matrix on the Biograph 64 TruePoint (2-dimensional, 2 iterations, 8 subsets) and in a 200 × 200 matrix on the Biograph mCT (3-dimensional, 2 iterations, 24 subsets, gaussian filter 5.0). The

focal uptake of ^{68}Ga-PSMA-11 above the background level and not relation to physiologic uptake or known pitfalls was considered PSMA-positive signals (Calais *et al.*, 2018).

Design and screening of highly optimized generation-2.5 antisense oligonucleotides (ASOs)

The ASOs used in this study contained a full phosphorothioate backbone and a 10-base 2′-deoxynucleoside gap flanked by 2′-O-methyl (cMt)-modified nucleotides. The motif for the ASOs targeting the *MLXIPe* RNA tested was mmm-10-mmm, where m represents cMt modification and -10- represents the 10-base DNA gap. ASOs were synthesized and purified as described previously (Seth *et al*, 2008). A Negative control ASO (5'-GGCTACTACGCCGTCA-3') with the same chemistry, but matching no human transcripts, Control were included in each experiment to demonstrate the specificity of *MLXIPe* ASOs. These oligonucleotides were designed to exclude G-strings with four Gs or two sets of three Gs in a row to prevent non-antisense-mediated effects (Zhao *et al.*, 2016).

m6A RIP-seq and data analysis

Pre-analysis quality control was performed using FastQC (http://www.bioinformatics.babraham.ac.uk/projects/fastqc/) and RSeQC software (Chen *et al*, 2015) to ensure that raw data are in excellent condition and suitable for downstream analyses. Pair-end raw reads were aligned to the human reference genome (GRch37/hg19) using Tophat (Cui *et al*, 2017). Genome-wide coverage signals were represented in BigWig format to facilitate convenient visualization using the UCSC genome browser. Gene expression was

measured using RPKM (Reads Per Kilo-base exon per Million mapped reads) as described previously (Cui *et al.*, 2017). Correlation analyses between RNA expression were performed using Python and R scripts.

MTS assay

The One-step 3-(4,5-dimethylthiazol-2-yl)-5-(3-carboxymethoxyphenyl)-2-(4-sulfophenyl)-2H-tetrazolium (MTS) assays were performed according to manufacturer's instructions (Promega). Briefly, patients derived primary cells were plated in 96-well plates at a density of 2,000 cells per well. At the indicated times, 20 µl of CellTiter 96R AQ_{ueous} Solution Reagent (Promega) was added to each well, and incubated for 2 h at 37 ºC in the cell incubator and then was measured in a microplate reader at 490 nm.

Row data of western blots were shown in Supplementary Figure 6.

Reference

Calais J, Kishan AU, Cao M, Fendler WP, Eiber M, Herrmann K, Ceci F, Reiter RE, Rettig MB, Hegde JV *et al* (2018) Potential Impact of (68)Ga-PSMA-11 PET/CT on the Planning of Definitive Radiation Therapy for Prostate Cancer. *J Nucl Med* 59: 1714-1721

Chen K, Luo GZ, He C (2015) High-Resolution Mapping of N(6)-Methyladenosine in Transcriptome and Genome Using a Photo-Crosslinking-Assisted Strategy. *Methods Enzymol* 560: 161-185

Cui Q, Shi H, Ye P, Li L, Qu Q, Sun G, Sun G, Lu Z, Huang Y, Yang CG *et al* (2017) m(6)A RNA Methylation Regulates the Self-Renewal and Tumorigenesis of Glioblastoma Stem Cells. *Cell Rep* 18: 2622-2634

Seth PP, Siwkowski A, Allerson CR, Vasquez G, Lee S, Prakash TP, Kinberger G, Migawa MT, Gaus H, Bhat B *et al* (2008) Design, synthesis and evaluation of constrained methoxyethyl (cMOE) and constrained ethyl (cEt) nucleoside analogs. *Nucleic Acids Symp Ser (Oxf)*: 553-554

Wen S, Wei Y, Zen C, Xiong W, Niu Y, Zhao Y (2020) Long non-coding RNA NEAT1 promotes bone metastasis of prostate cancer through N6-methyladenosine. *Mol Cancer* 19: 171

Zhao Y, Ding L, Wang D, Ye Z, He Y, Ma L, Zhu R, Pan Y, Wu Q, Pang K *et al* (2019) EZH2 cooperates with gain-of-function p53 mutants to promote cancer growth and metastasis. *EMBO J* 38

Zhao Y, Wang L, Ren S, Wang L, Blackburn PR, McNulty MS, Gao X, Qiao M, Vessella RL, Kohli M *et al* (2016) Activation of P-TEFb by Androgen Receptor-Regulated Enhancer RNAs in Castration-Resistant Prostate Cancer. *Cell Rep* 15: 599-610

Fig S1

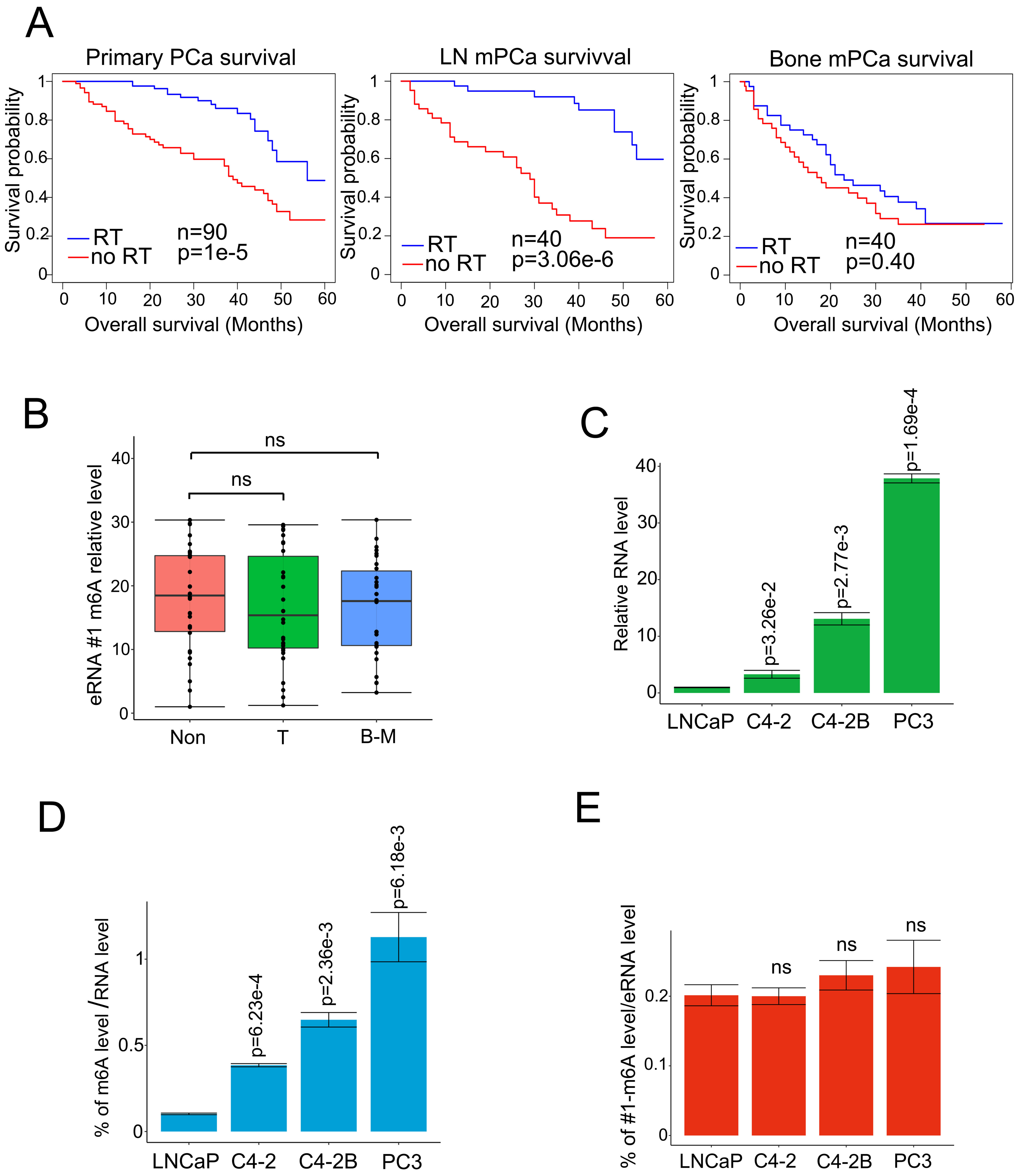

A
Primary PCa survival
LN mPCa survivval
Bone mPCa survival
Survival probability
Overall survival (Months)
RT
no RT
n=90
p=1e-5
n=40
p=3.06e-6
n=40
p=0.40
B
eRNA #1 m6A relative level
ns
ns
Non
T
B-M
C
Relative RNA level
p=3.26e-2
p=2.77e-3
p=1.69e-4
LNCaP
C4-2
C4-2B
PC3
D
% of m6A level /RNA level
p=6.23e-4
p=2.36e-3
p=6.18e-3
LNCaP
C4-2
C4-2B
PC3
E
% of #1-m6A level/eRNA level
ns
ns
ns
LNCaP
C4-2
C4-2B
PC3

Fig S2

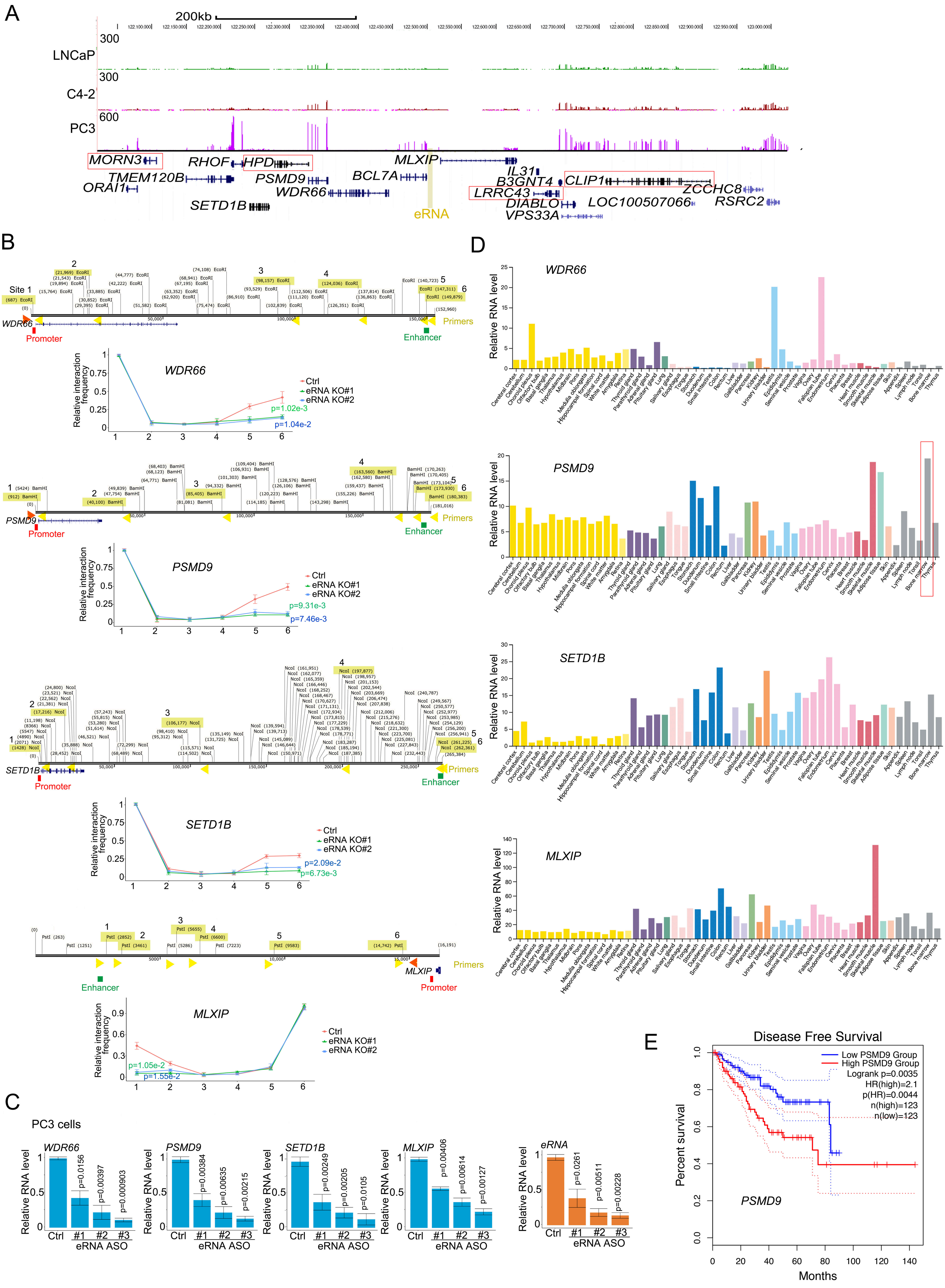
A
200kb
LNCaP
C4-2
PC3
MORN3
RHOF
HPD
MLXIP
TMEM120B
PSMD9
BCL7A
IL31
B3GNT4
CLIP1
ORAI1
WDR66
LRRC43
ZCCHC8
SETD1B
DIABLO
LOC100507066
RSRC2
VPS33A
eRNA
B
Promoter
Enhancer
Primers
WDR66
PSMD9
SETD1B
MLXIP
Relative interaction frequency
Ctrl
eRNA KO#1
eRNA KO#2
p=1.02e-3
p=1.04e-2
p=9.31e-3
p=7.46e-3
p=2.09e-2
p=6.73e-3
p=1.05e-2
p=1.55e-2
C
PC3 cells
Relative RNA level
eRNA ASO
eRNA
D
Relative RNA level
E
Disease Free Survival
Low PSMD9 Group
High PSMD9 Group
Logrank p=0.0035
HR(high)=2.1
p(HR)=0.0044
n(high)=123
n(low)=123
Percent survival
Months

Fig S3

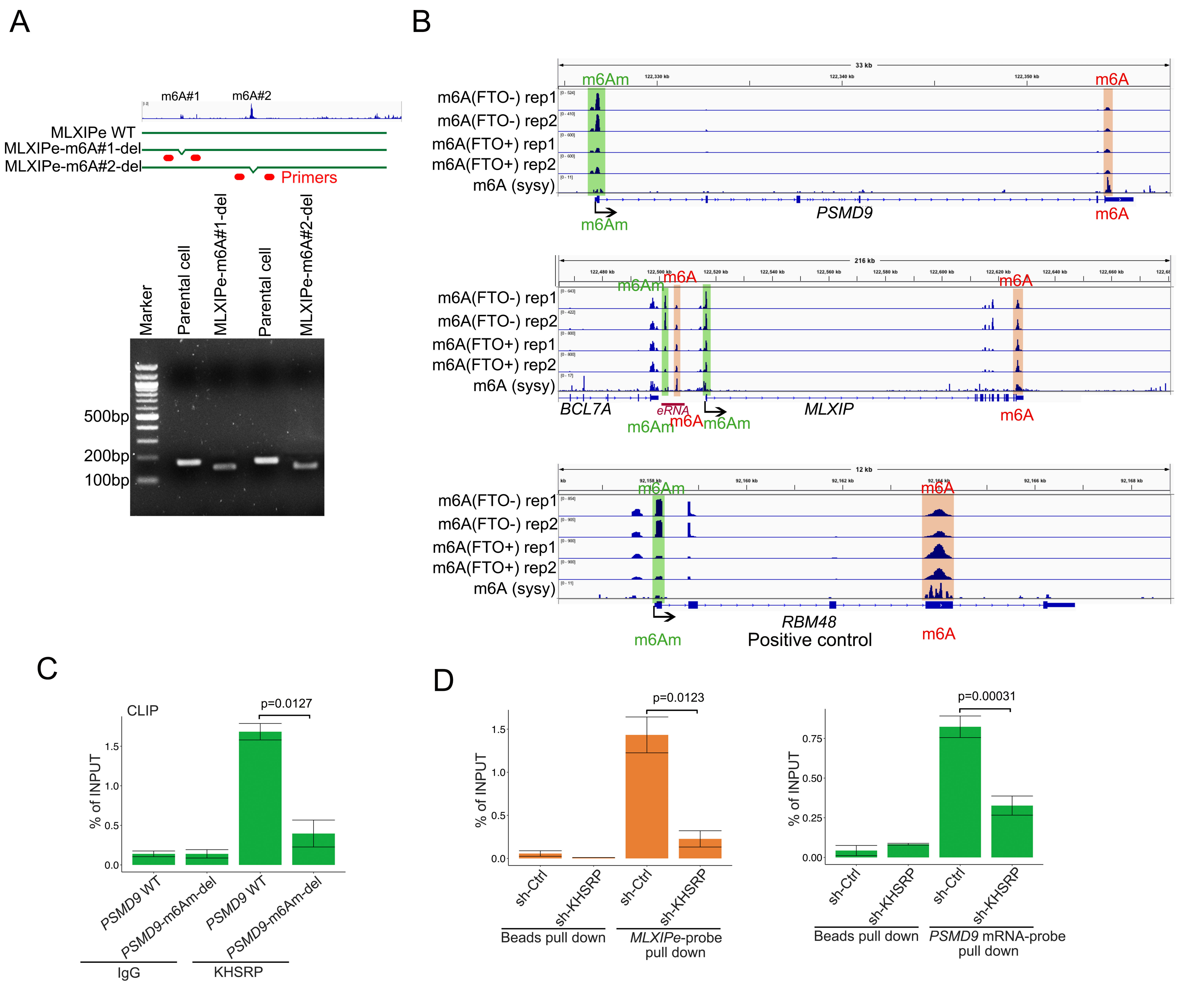

A
m6A#1
m6A#2
MLXIPe WT
MLXIPe-m6A#1-del
MLXIPe-m6A#2-del
Primers
Marker
Parental cell
MLXIPe-m6A#1-del
Parental cell
MLXIPe-m6A#2-del
500bp
200bp
100bp
B
33 kb
m6Am
m6A
m6A(FTO-) rep1
m6A(FTO-) rep2
m6A(FTO+) rep1
m6A(FTO+) rep2
m6A (sysy)
PSMD9
216 kb
BCL7A
eRNA
MLXIP
12 kb
RBM48
Positive control
C
CLIP
p=0.0127
% of INPUT
PSMD9 WT
PSMD9-m6Am-del
IgG
KHSRP
D
p=0.0123
p=0.00031
sh-Ctrl
sh-KHSRP
Beads pull down
MLXIPe-probe pull down
PSMD9 mRNA-probe pull down

Fig S4

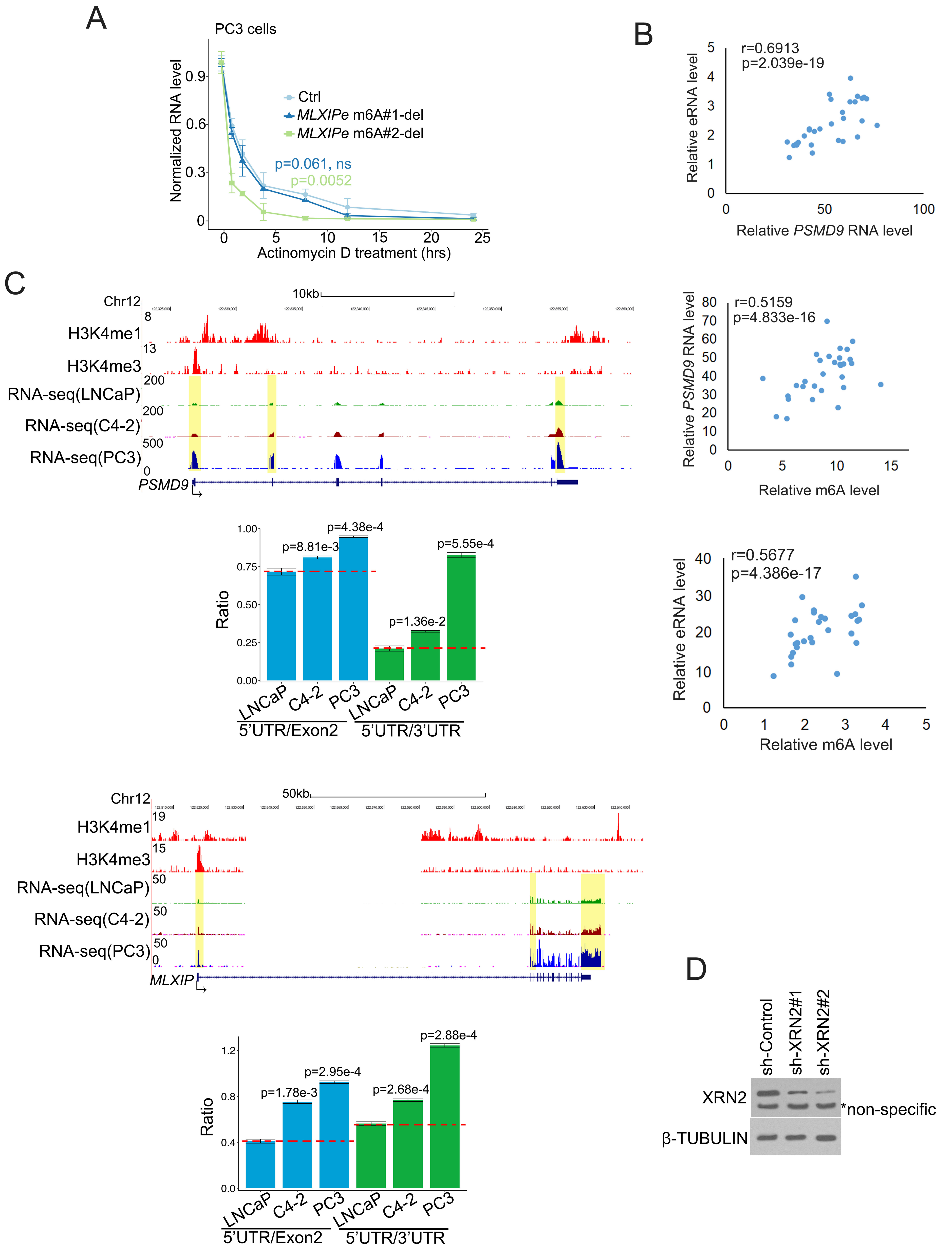

A
PC3 cells
Normalized RNA level
Actinomycin D treatment (hrs)
Ctrl
MLXIPe m6A#1-del
MLXIPe m6A#2-del
p=0.061, ns
p=0.0052
B
r=0.6913
p=2.039e-19
Relative eRNA level
Relative PSMD9 RNA level
r=0.5159
p=4.833e-16
Relative PSMD9 RNA level
Relative m6A level
r=0.5677
p=4.386e-17
Relative eRNA level
Relative m6A level
C
Chr12
10kb
H3K4me1
H3K4me3
RNA-seq(LNCaP)
RNA-seq(C4-2)
RNA-seq(PC3)
PSMD9
Ratio
p=8.81e-3
p=4.38e-4
p=1.36e-2
p=5.55e-4
LNCaP
C4-2
PC3
5'UTR/Exon2
5'UTR/3'UTR
Chr12
50kb
H3K4me1
H3K4me3
RNA-seq(LNCaP)
RNA-seq(C4-2)
RNA-seq(PC3)
MLXIP
Ratio
p=1.78e-3
p=2.95e-4
p=2.68e-4
p=2.88e-4
5'UTR/Exon2
5'UTR/3'UTR
D
sh-Control
sh-XRN2#1
sh-XRN2#2
XRN2
*non-specific
β-TUBULIN

Fig S5

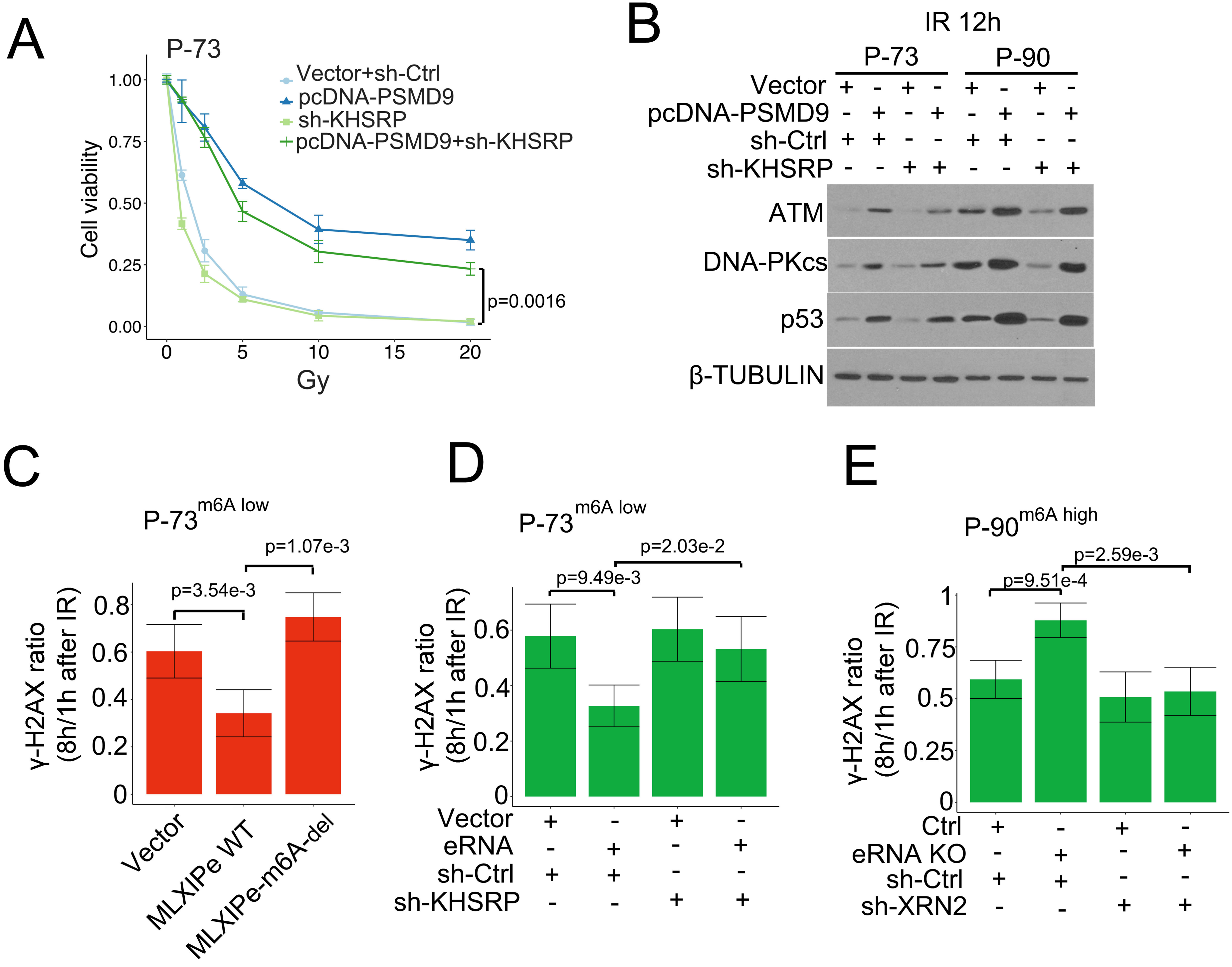
A
P-73
Vector+sh-Ctrl
pcDNA-PSMD9
sh-KHSRP
pcDNA-PSMD9+sh-KHSRP
Cell viability
1.00
0.75
0.50
0.25
0.00
0
5
10
15
20
Gy
p=0.0016
B
IR 12h
P-73
P-90
Vector + - + - + - + -
pcDNA-PSMD9 - + - + - + - +
sh-Ctrl + + - - + + - -
sh-KHSRP - - + + - - + +
ATM
DNA-PKcs
p53
β-TUBULIN
C
P-73 m6A low
p=3.54e-3
p=1.07e-3
γ-H2AX ratio (8h/1h after IR)
0.8
0.6
0.4
0.2
0
Vector
MLXIPe WT
MLXIPe-m6A-del
D
P-73 m6A low
p=9.49e-3
p=2.03e-2
γ-H2AX ratio (8h/1h after IR)
0.6
0.4
0.2
0
Vector + - + -
eRNA - + - +
sh-Ctrl + + - -
sh-KHSRP - - + +
E
P-90 m6A high
p=9.51e-4
p=2.59e-3
γ-H2AX ratio (8h/1h after IR)
1
0.75
0.5
0.25
0
Ctrl + - + -
eRNA KO - + - +
sh-Ctrl + + - -
sh-XRN2 - - + +

Fig. S6

S6A related to Fig 1G

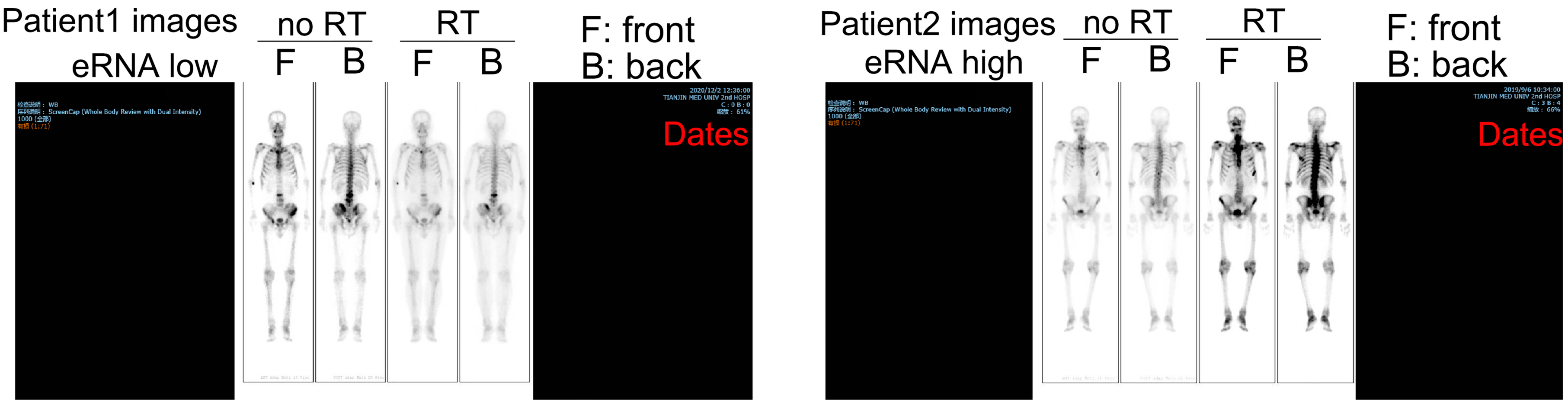

S6B

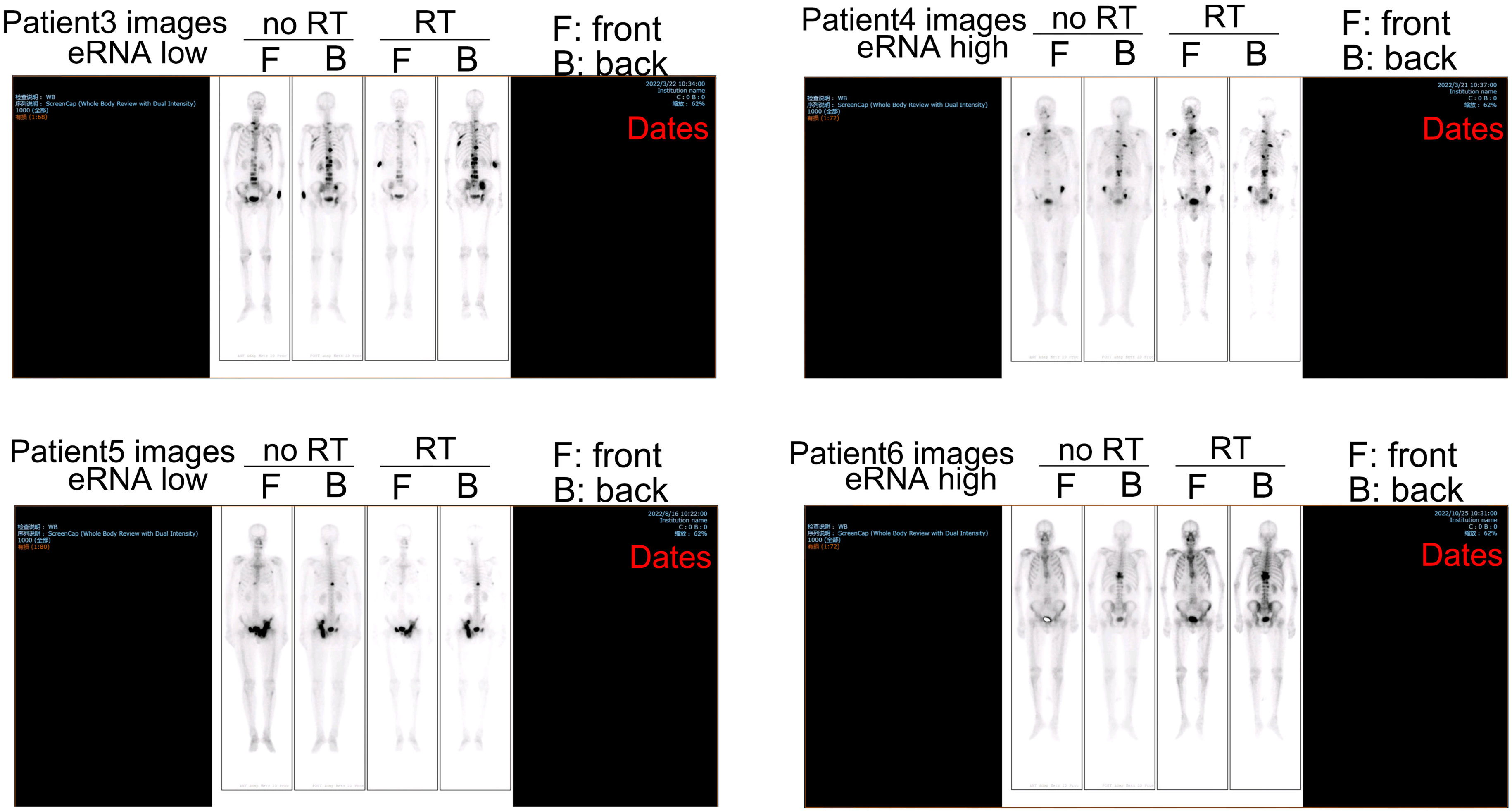

Fig.S7

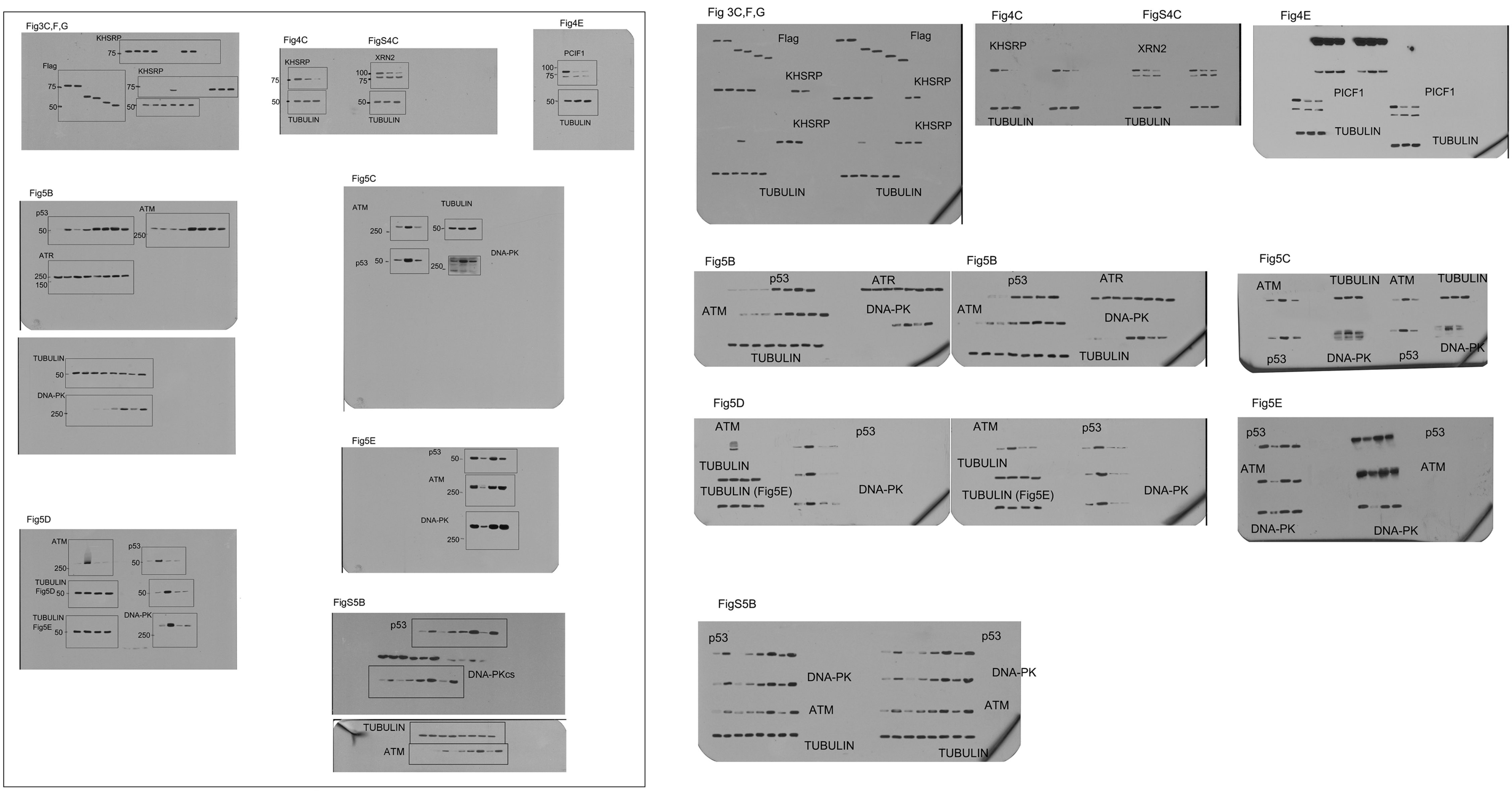

Images used in Article
